# Evaluating preschool linear growth velocities: an interim reference illustrated in Nepal

**DOI:** 10.1017/S1368980023002409

**Published:** 2023-12

**Authors:** Swetha Manohar, Elizabeth Colantuoni, Andrew Lucian Thorne-Lyman, Binod Shrestha, Ramesh Kant Adhikari, Angela KC, Abhigyna Bhattarai, Keith Parker West

**Affiliations:** 1 Center for Human Nutrition, Department of International Health, Johns Hopkins Bloomberg School of Public Health, Baltimore, MD, USA; 2 Department of Biostatistics, Johns Hopkins Bloomberg School of Public Health, Baltimore, MD, USA; 3 PoSHAN Study Team, Johns Hopkins University, Patan Dhoka, Lalitpur, Nepal; 4 Department of Child Health, Institute of Medicine, Tribhuvan University, Nepal; 5 Global Food Ethics & Policy Program, Johns Hopkins School of Advanced International Studies, Washington, DC 20036, USA; 6 Blitz Media Pvt. Ltd., Tripureshwor, Maharajgunj, Kathmandu, Nepal

**Keywords:** Linear growth velocity, Growth faltering, Anthropometry, Growth velocity reference, Nepal

## Abstract

**Objective:**

An annualised linear growth velocity (LGV) reference can identify groups of children at risk of growing poorly. As a single velocity reference for all preschool ages does not exist, we present an interim tool, derived from published, normative growth studies, for detecting growth faltering, illustrating its use in Nepali preschoolers.

**Design:**

The WHO Child Growth Velocity Standard was adapted to derive 12-month increments and conjoined to the Tanner-Whitehouse Height Velocity Reference data yielding contiguous preschool linear growth annualised velocities. Linear restricted cubic spline regressions were fit to generate sex-specific median and standard normal deviate velocities for ages 0 through 59 months. LGV *Z*-scores (LGVZ) were constructed, and growth faltering was defined as LGVZ < –2.

**Setting:**

Use of the reference was illustrated with data from Nepal’s *Tarai* region.

**Participants:**

Children contributing the existing growth references and a cohort of 4276 Nepali children assessed from 2013 to 2016.

**Results:**

Fitted, smoothed LGV reference curves displayed monotonically decreasing 12-month LGV, exemplified by male/female annual medians of 26·4/25·3, 12·1/12·7, 9·1/9·4, 7·7/7·8 and 7/7 cm/years, starting at 0, 12, 24, 36 and 48 months, respectively. Applying the referent, 31·1 %, 28·6 % and 29·3 % of Nepali children <6, 6–11 and 12–23 months of age, and ∼6 % of children 24–59 months, exhibited growth faltering. Under 24 months, faltering velocities were more prevalent in girls (34·4 %) than boys (25·3 %) (*P* < 0·05) but comparable (∼6 %) in older preschoolers.

**Conclusions:**

A LGV reference, concatenated from extant data, can identify preschool groups at-risk of growth faltering. Application and limitations are discussed.

## Graphical abstract

**Figure:**
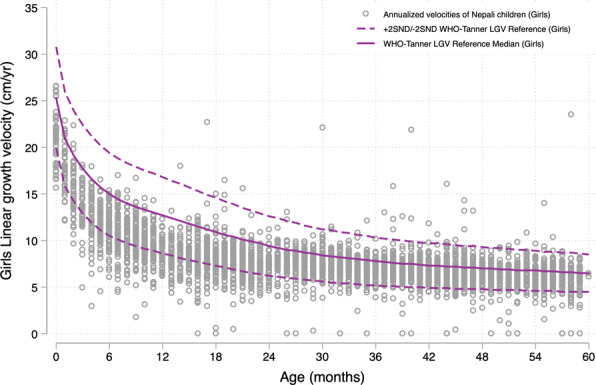

A reduction in preschool linear growth stunting by 40 % is a Sustainable Development Goal to which low-middle income country governments are committed by 2030^(1)^. Progress has been noteworthy, reflected by a steady, yet inconsistent, decline in early childhood stunting across regions of the world^(2)^. For example, across South Asia the prevalence of stunting (defined as length or height for age < −2 standard normal deviates (SND) or *Z*-scores) decreased from ∼48 % to ∼33 % between 2000 and 2018^(2,3)^, a trend which, if sustained, predicts countries in the region will not achieve the Sustainable Development Goal targets by 2030, with the exception of Bangladesh^(4)^. More recent national surveys suggest the initial rapid downward trend has slowed and in some countries levelled off^(3,5,6)^, with similar patterns emerging in other regions^(7)^. While causes behind the pause remain unclear, the disruption challenges countries to further innovate in their efforts to reduce childhood stunting and its apparent health, nutrition and economic consequences through adulthood^(8,9)^.

Tracking progress towards reducing stunting relies primarily on population-based national surveys, which generate prevalence estimates and offer opportunities for case–control analyses of risk factors associated with a short attained length or height for age (L/HAZ) in relation to the cross-sectional WHO Child Growth Standard^(10)^. By design, surveys can only quantify the burden at a point in time and, typically, compare risk factors between those already stunted *v*. not. Cross-sectional assessments are unable to detect age of onset, severity or duration of decline in growth. Nor are they able to detect time-dependent risk factors that could identify population groups at risk of subsequent growth faltering^(11)^, all of which may predispose children to becoming stunted. Examined this way, children above −2 L/HAZ who are nonetheless experiencing subnormal growth rates are misclassified as normal in case–control analyses, leading to a dilution of risk factor effect sizes. Second, children growing at low velocity can be considered ‘cases’ (at any length/height for age) whose age, sex, socio-economic, cultural or other risk characteristics may help to subsequently identify *groups* at risk to monitor and possibly intervene to preserve normal growth and reduce incident stunting *in a population*.

Active growth faltering, reflected by a subnormal velocity over a consequential period of time, may be as, or more, frequent than attained stunting^(12)^. However, the ability to assess its extent necessitates longitudinal evaluation against a normative referent of age-sex-specific growth velocities. Growth velocities have typically been studied over intervals of weeks to several months^(13,14)^. While shorter intervals are clinically relevant, an annual interval offers distinct advantages over shorter periods for identifying groups at risk by accommodating fluctuations in growth associated with seasonality, minimising effects of measurement error on velocity estimation and reducing costs of obtaining multiple shorter increments. Further, although an annual increment necessitates two paired measurements taken approximately the same month a year apart, the paired assessments can occur during any month of the year without affecting the validity of an annualised velocity measurement.

Notwithstanding this potential, as well as logistical and cost elements, a major constraint to annual linear growth velocity (LGV) assessment is the absence of continuous, sex-age-specific reference curves starting at month of birth and extending, month-by-month, through the preschool years (up to 59 months). While arguably needed, development of a multi-country, normative, annual growth velocity study and reference will be costly and likely require a decade or longer to plan, conduct, analyse and disseminate^(15)^. As an interim alternative, a limited number of normative child growth studies, with either annual growth increments or increments amenable to being annualised, exist across the preschool age spectrum^(16–19)^. Under a well-established premise that variation in linear growth is affected mainly by environment (*v*. genetic variation), such that different child populations grow comparably in supportive environs^(20–24)^, we present a growth reference concatenated from extant normative studies. We share sex-specific linear growth curves with proposed SND, covering the entire preschool age range by adapting and combining two well-published referents: the WHO Child Growth Standard for Length Velocity^(17)^ to generate normalised, 12-month velocity curves starting at birth through 12 months and the Tanner-Whitehouse Height Velocity reference^(16)^ that offers spaced 12-month increment distributions of children in the UK, starting from 13 through 59 months of age. We illustrate the utility of this approach by evaluating annualised growth velocities in a cohort of 4276 preschool-aged children in the *Tarai* (Southern Plains) of Nepal and discuss the epidemiological and potential intervention value as well as limitations of this approach.

## Subjects and methods

### Development of a 12-month linear growth velocity reference

In search of candidate normative growth data, we reviewed the literature for existing, fully documented, LGV distributions reportedly derived from healthy, preschool-aged child populations and assessed their suitability for our use in deriving an annualised reference.

#### Inclusion criteria for linear growth velocity references

Referent growth studies were eligible for consideration if they: (1) reported 3-month, 6-month or 12-month length or height increments, with preference for the last; (2) included corresponding measures of statistical uncertainty (i.e. SD) for velocity estimates; (3) were available by month of age starting from 0 to 59 months; (4) were sex-specific and (5) were conducted in generally supportive dietary/nutritional, health care, family and environmental conditions. Our review of existing growth references with fit-for-purpose potential is described in Supplemental Table 1 and Section I of the Online Supporting Material. There was no single study that reported 12-month increments by each starting month of age throughout the preschool years, leading to a need to combine and model data across studies.

#### Selection of linear growth velocity references

We selected references from which annual velocities could be derived and age-specific distributions smoothly conjoined to reflect a generally healthy growth trajectory, albeit drawn from different populations^(16,25)^. Among those identified, the WHO 2006 Child Growth Standard for Length Velocity^(17)^ (henceforth referred to as the WHO Child Growth Standard) and 1965 Tanner-Whitehouse Height Velocity Reference (henceforth referred to as the Tanner Reference)^(16)^ were found to fit these criteria.

The WHO Child Growth Standard presents 6-monthly LGV distributions by sex among cohorts of healthy children from six countries (Brazil, Ghana, India, the USA, Oman and Norway) participating from birth comprising the population-based Multicentre Growth Reference Study (MGRS), conducted in the field from 1997 to 2003^(17)^. In the MGRS, children were followed monthly during the 1^st^, and bi-monthly during the second, years of life, with 6-monthly linear growth rates summarised for each month of age from 0 to 18 months, inclusive (ending at age 24 months)^(17)^. For this analysis, we estimated average 12-month growth rates for consecutive ages 0–12 months by summing end-to-end 6-month rates. For example, the median 6-month linear growth rate, in cm, from month 0 (i.e., birth month through 5th month, or as reported by WHO as the 0–6-month interval), was added to the 6th through 11th month median increment (reported as the 6–12-month interval) to approximate a 0-to-12-month median velocity in cm/year (see online Supplemental Table 2, ‘Annualised Length Velocity (LV)’ for formula). This process was repeated, ending with a 12–24 month velocity estimate. Standard deviations for derived annualised length velocities were estimated by the formula: Σ{(Median+(−1 SD) + (1 SD-Median)}/2, which averages the values for −1 sd and + 1 sd values reported for the median in the original WHO Standard curve that is approximately normal (formula listed in Supplemental Table 2, ‘sd (Annualised LV)’).

The Tanner Reference was developed from measurements of children living in Central London and randomly selected from records of families having regular health checks at the University of London’s Institute of Child Health primarily during 1954. Study children belonged to the Child Study Center Group, assumed to represent urban British children^(25)^. Tabular data provided 12-month growth increments for children aged 2 months to 18 years, presented at 3 month intervals between ages 2 to 22 months and 6-month intervals from 27 months of age onwards^(16)^. These data are published and publicly available^(16)^. This data structure required us to interpolate median and SD (see online Supplemental Table 2, ‘Annualised Height Velocity (HV)’ and ‘sd (Annualised HV)’ for formula) for intra-interval monthly ages, allowing estimation of continuous 12-month velocity distributions for each month of age from 13 to 59 months of age. Expanded descriptions of the WHO Child Growth Standard and the Tanner Reference are given in Supplemental Table 1.

### Construction of a single linear growth velocity reference

We combined the WHO Child Growth Standard and Tanner Reference to form a single, annual LGV reference based on criteria we set forth and their extensive presence in the growth literature^(26)^. This approach is supported by studies that have shown minimal variation in linear growth rates and height-for-age distributions among children living in generally supportive socio-economic conditions, regardless of geographic location and genotype^(17,20,23,24,27–29)^. For example, the WHO study noted ∼1–7 % variability in heights across its six country locations and that on average height of children differed by only ∼3 %^(30)^. With respect to the single site from which Tanner data emerged, comparison of median and accompanying SD data describing annual linear growth with growth data generated from the Zurich Longitudinal Study of Growth for the purpose of establishing a velocity reference revealed highly comparable data for both girls and boys^(18)^ (online Supplemental Tables 3(a) and (b)).

Using the above approaches, we derived a single growth velocity reference by combining approximated 12-month velocities from the WHO Child Growth Standard for the age range birth to 12 months and the Tanner Reference for ages 13–59 months. The derived length/height distributions in tabular format are detailed in online Supplemental Table 4. As the WHO and Tanner distributions are being considered normative references, we propose the associated standard deviations be considered equivalent to SND, and thus providing the basis for *Z*-score estimates, displayed out to −3 to +3 SND from the median, and the basis for evaluating in-country growth velocity distributions. The CV for each 12-month increment by sex was estimated based on an assumption of normality of both original reference velocity distributions. Estimates of the sex-specific and sex combined velocities at each SND from the median were plotted against age to examine and affirm patterns of monotonicity and non-linearity.

**Fig. 1:**
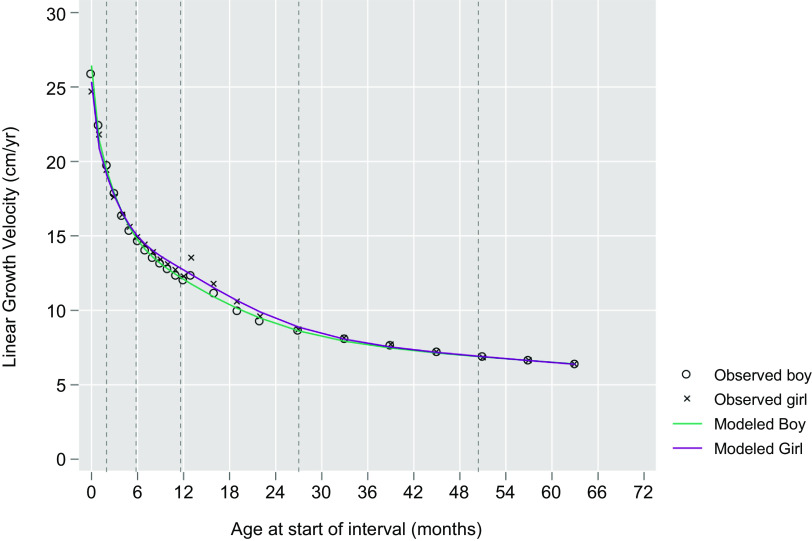
WHO-Tanner growth velocity reference curve: plotted medians and modelled 12- month velocities by sex from birth (month 0) through 59 months of age*^†^. *Age at the start of interval. ^†^Generated using a cubic-restricted spline model with knots at 2, 6, 12, 27 and 50 (dotted lines) months of age: 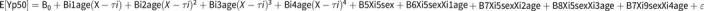

**Fig. 2:**
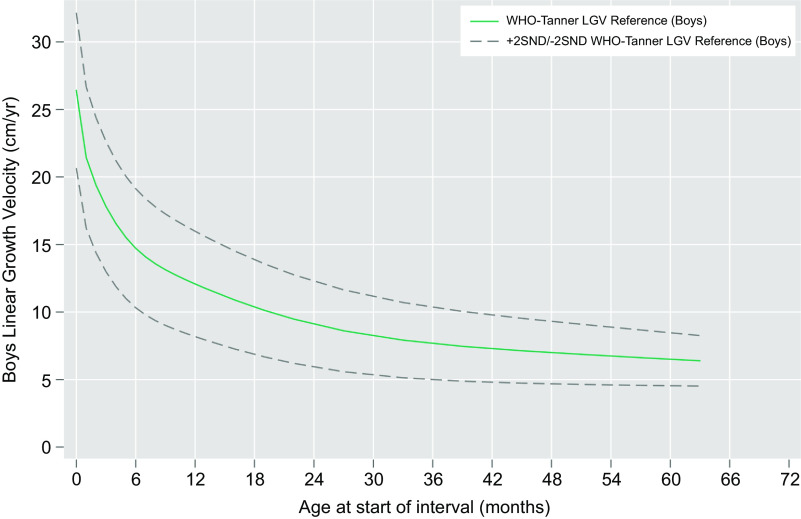
WHO-Tanner modelled linear growth velocity reference curve for boys from birth (month 0) through 59 months of age at the start of the growth interval (median + 2 *Z*-scores)

Linear regression models were fit to estimate median and variance in height velocities as a function of age, sex and the interaction of age and sex (Figs. 1 and 2). A sex-combined curve was also fit (online Supplemental Figs. 1 and 2). Age was modelled using restricted cubic splines with pre-specified knots at 2, 6, 12, 27 and 50 months. Wald tests were used to determine if velocities and their relationship with age differed by sex. Predicted values from the models were obtained and serve as the final values derived for each sex-specific combined velocity reference curve. The final models were fit excluding the interaction of age and sex to obtain sex-specific estimates.

### Application of the reference: Nepal study population

We demonstrate the use and interpretation of this novel interim reference with anthropometric data collected annually between 2013 and 2016 from a representative sample of children ≤71 months of age living in households across the southern plains (*Tarai*) of Nepal. The study sample comprised a portion of a larger nationally representative, multi-year project that assessed linkages between agriculture, food security and nutrition^(31)^. Bordering India, the *Tarai* region is flat compared with hilly and mountainous regions in Nepal, housing approximately half of Nepal’s population, nearly ∼80 % of which is engaged in agriculture as smallholder farmers^(32)^. The region is, nonetheless, endemically undernourished reflected in preschool children by a high prevalence of both stunting (34 %) and wasting (18·9 %) in 2016^(5)^ and where close to half of children (and women) have anaemia^(33–35)^, attributed to dietary inadequacy, infectious diseases and social, cultural and economic inequities^(13,36)^.

Seven Village Development Committees (sub-districts) were systematically selected following a random start from a complete list of *Tarai* Village Development Committees ordered from west to east. Within each Village Development Committee, 3 of 9 administrative wards ordered by population size were systematically sampled following a random start, resulting in 21 selected wards. Eligibility for the study was based on a child under-5 year of age residing in consenting households in sampled wards. The initial sample of households was visited each mid-year as were new households in selected wards with preschool children. Ethical approval for each PoSHAN survey was obtained from the Nepal Health Research Council, an autonomous body under the Ministry of Health and Population of the Government of Nepal and the Institutional Review Board at the Johns Hopkins Bloomberg School of Public Health, Baltimore, Maryland.

#### Anthropometry

Children were assessed through 2016 or up to (<) 71 months of age. Measurements of recumbent length, <24 months, and standing height, >24 months of age, were read to the nearest 0·1 cm in triplicate using ShorrBoards® (Weigh and Measure, LLC), with the median considered the final value. Boards were calibrated weekly using standard length rods. Other measurements included weight and mid-upper arm circumference, not addressed further in this article. Anthropometry was performed by field staff trained and standardised annually, demonstrating a relative technical error of measurement of <2 % for length or height^(31)^. During fieldwork, measurements were independently repeated on 10 % of children by quality control staff.

#### Estimating linear growth velocity *Z*-scores

A total of 4276 children contributed LGV data, based on having 1, 2 or 3 paired measurements of length or heights ∼12 months apart. Difference in height (Δ height) was calculated by subtracting a previous length or height, at the outset of an interval, from an end-of-interval value and annualised by dividing the difference in height by the number of days between assessments and multiplying by 365·25 d (see online Supplemental Table 2, ‘Δ Height’ for formula used). Overall, the study’s loss to follow-up was low (3 %). A subsample of 612 children lacked paired measurements and were excluded from analysis. Compared with included children, those excluded were slightly older, more likely male and born to less educated mothers (data not shown). Surprisingly, children of excluded mothers were also noted to have a lower proportion of stunting (29·8 % *v*. analytic sample: 36·7 %) and underweight (loss to follow-up: 35·6 % *v*. analytic sample: 39·6 %). No differences were noted in the proportion wasted or with recent report of diarrhoea.

The final analytic sample comprised 4276 children and 8356 growth intervals. HAZ beyond ± 6 SND (*n* 11, <1 % of sample) and child delta height (Δ height) <0 cm/year were converted to missing values (*n* 15, <1 % of sample). Children with missing length/height data (*n* 68) or 1·3 % of the sample were excluded.

We compared velocity data from the Nepali sample to the derived WHO-Tanner reference to derive linear growth velocity *Z*-score (LGVZ) values using the standard formula for computing *Z*-scores (see online Supplemental Table 2, ‘LGVZ**’** for formula). Three velocities exceeded ± 10 SND and were excluded, consistent with the published velocity literature^(17,37)^. Linear growth faltering was defined as LGVZ < -2 against the derived WHO-Tanner velocity reference adopting the same convention to classify static distributions of stunting (HAZ < -2)^(38)^.

Child ages at the outset of annualised intervals were stratified into six groups. The newborn and infancy period was the <6 months age group, a period of fastest postnatal growth when infants are expected to be exclusively breastfed, following which was the 6–11·9 months age group, when children are being introduced to complementary foods and growth velocity remains high. Thereafter, velocities were grouped by each year of age: 12–23·9 months, when velocity markedly slows and children’s diets continue to evolve to a family diet, and 24–35·9, 36–47·9 and 48–59·9 months, representing intervals of more stable linear growth^(39,40)^. Given the multi-year follow-up design of our study in Nepal, as children aged, many contributed one 12-month growth increment to 2 or more intervals, retaining independence of velocity data within each age stratum.

Confidence intervals around mean LGVZ, and consequent percentages of growth velocities classified as faltering (< −2 LGVZ), were estimated accounting for clustering. Differences in mean velocity *Z*-scores and proportions faltering by sex within each age stratum were evaluated for statistical significance by a paired *t*-test and Pearson’s chi-square test, respectively. The patterns of overlap in stunting and linear growth faltering experienced were assessed by age. These estimates reflect the proportion of children stunted (HAZ < -2) or not at the beginning of an age interval and the proportion of children who experience faltered growth (LGVZ < -2) or not during that 12-month interval of age. All analyses were conducted using STATA 14 SE.

## Results

**Fig. 3:**
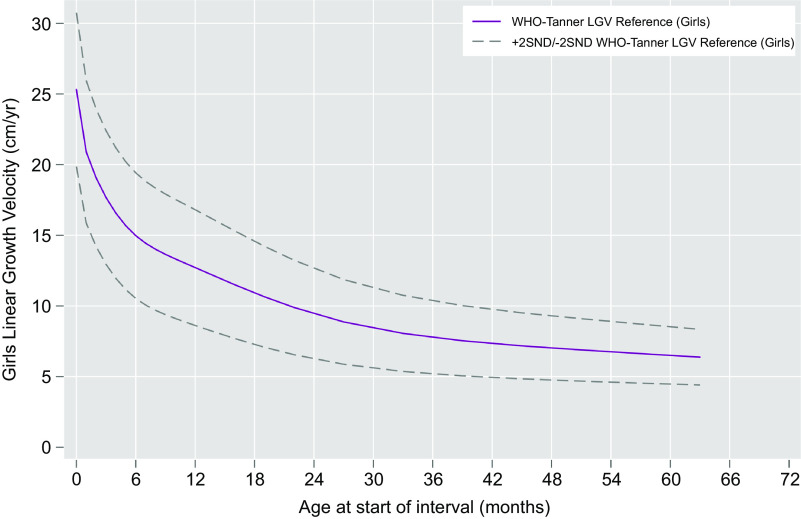
WHO-Tanner modelled linear growth velocity reference curve for girls from birth (month 0) through 59 months of age at the start of the growth interval (median + 2 *Z*-scores)

Figure 3 plots by sex the modelled median, 12-month growth velocities by starting month of age through infancy from the WHO Child Growth Standard and from the Tanner Reference, measured quarterly and semi-annual median velocities through 63 months (to enable estimation of a velocity at 59 months). Superimposed are the modelled median sex-specific growth velocity curves obtained by restricted cubic spline regression analysis (within indicated ages of prespecified knots), visually demonstrating a monotonic decline and degree of goodness-of-fit of curves to the data. Figures 1 and 2 present the same median velocities with associated SND, representing *Z*-scores, starting from the month of birth (‘0’) through 59 months of age for boys and girls, respectively. Differences in velocities between boys and girls were noted in the WHO-Tanner Reference between the ages of 7–27 months. Expectedly, SND intervals narrow as growth rates decline with age. Table 1 displays the modelled median and SND data for each sex-age-specific interval through 59 months of age. Supplemental Table 4 presents the unmodelled derived estimates for the median and SND data for each sex and the same age range.

**Table 1:** Reference 12-month linear growth increments by sex and age from the modelled derived WHO-Tanner linear growth velocity curve^*,†^

	Length/height increments in cm/year													
Age	Boys							Girls						
Interval	−3 SND^‡^	−2 SND^‡^	−1 SND^‡^	Median^§^	1 SND^‡^	2 SND^‡^	3 SND^‡^	−3 SND^‡^	−2 SND^‡^	−1SND^‡^	Median^§^	1 SND^‡^	2 SND^‡^	3 SND^‡^
0–12 mo	18·8	20·7	22·7	26·4	30·2	32·2	34·0	18·2	19·9	21·8	25·3	28·9	30·8	32·5
1–13 mo	14·5	16·2	18·0	21·4	24·9	26·7	28·3	14·3	15·8	17·6	20·9	24·2	25·9	27·5
2–14 mo	12·8	14·4	16·1	19·4	22·6	24·4	26·0	12·6	14·2	15·9	19·1	22·2	23·9	25·5
3–15 mo	11·5	13·0	14·7	17·8	21·0	22·6	24·1	11·4	12·9	14·6	17·7	20·8	22·4	23·9
4–16 mo	10·4	11·9	13·5	16·5	19·6	21·2	22·7	10·5	11·9	13·5	16·6	19·6	21·2	22·6
5–17 mo	9·6	11·0	12·6	15·5	18·5	20·1	21·5	9·7	11·1	12·7	15·7	18·6	20·2	21·6
6–18 mo	8·9	10·3	11·9	14·7	17·6	19·1	20·5	9·1	10·5	12·1	15·0	17·9	19·4	20·8
7–19 mo	8·4	9·8	11·3	14·1	16·9	18·4	19·7	8·7	10·1	11·6	14·4	17·3	18·8	20·2
8–20 mo	8·0	9·4	10·8	13·6	16·3	17·8	19·1	8·3	9·7	11·2	14·0	16·8	18·3	19·7
9–21 mo	7·7	9·0	10·4	13·1	15·8	17·3	18·6	8·0	9·4	10·9	13·6	16·4	17·9	19·3
10–22 mo	7·4	8·7	10·1	12·8	15·4	16·8	18·1	7·8	9·1	10·6	13·3	16·1	17·5	18·9
11–23 mo	7·2	8·4	9·8	12·4	15·0	16·4	17·6	7·5	8·9	10·3	13·0	15·7	17·2	18·5
12–24 mo	7·0	8·2	9·5	12·1	14·6	16·0	17·2	7·3	8·6	10·0	12·7	15·4	16·8	18·1
13–25 mo	6·7	7·9	9·3	11·8	14·3	15·6	16·8	7·1	8·4	9·8	12·4	15·0	16·4	17·7
14–26 mo	6·5	7·7	9·0	11·5	13·9	15·2	16·4	6·9	8·1	9·5	12·1	14·7	16·1	17·3
15–27 mo	6·3	7·5	8·8	11·2	13·6	14·9	16·0	6·7	7·9	9·3	11·8	14·3	15·7	16·9
16–28 mo	6·1	7·3	8·5	10·9	13·3	14·5	15·7	6·5	7·7	9·0	11·5	14·0	15·3	16·5
17–29 mo	5·9	7·1	8·3	10·6	13·0	14·2	15·3	6·3	7·5	8·8	11·2	13·6	14·9	16·1
18–30 mo	5·8	6·9	8·1	10·4	12·7	13·9	15·0	6·1	7·3	8·5	10·9	13·3	14·6	15·7
19–31 mo	5·6	6·7	7·9	10·1	12·4	13·6	14·7	5·9	7·1	8·3	10·6	13·0	14·2	15·4
20–32 mo	5·4	6·5	7·7	9·9	12·1	13·3	14·4	5·8	6·9	8·1	10·4	12·7	13·9	15·0
21–33 mo	5·3	6·3	7·5	9·7	11·9	13·0	14·1	5·6	6·7	7·9	10·1	12·4	13·5	14·6
22–34 mo	5·2	6·2	7·3	9·5	11·6	12·8	13·8	5·5	6·5	7·7	9·9	12·1	13·2	14·3
23–35 mo	5·0	6·1	7·2	9·3	11·4	12·5	13·5	5·4	6·4	7·5	9·7	11·8	12·9	14·0
24–36 mo	4·9	5·9	7·0	9·1	11·2	12·3	13·3	5·2	6·2	7·4	9·4	11·5	12·6	13·6
25–37 mo	4·8	5·8	6·9	8·9	11·0	12·0	13·0	5·1	6·1	7·2	9·2	11·3	12·4	13·4
26–38 mo	4·7	5·7	6·8	8·8	10·8	11·8	12·8	5·0	6·0	7·0	9·0	11·0	12·1	13·1
27–39 mo	4·6	5·6	6·6	8·6	10·6	11·6	12·6	4·9	5·9	6·9	8·9	10·8	11·9	12·8
28–40 mo	4·5	5·5	6·5	8·5	10·4	11·5	12·4	4·8	5·8	6·8	8·7	10·6	11·6	12·6
29–41 mo	4·5	5·4	6·4	8·3	10·3	11·3	12·2	4·8	5·7	6·7	8·6	10·4	11·4	12·3
30–42 mo	4·4	5·3	6·3	8·2	10·1	11·1	12·1	4·7	5·6	6·6	8·4	10·3	11·2	12·1
31–43 mo	4·4	5·3	6·2	8·1	10·0	11·0	11·9	4·6	5·5	6·5	8·3	10·1	11·1	11·9
32–44 mo	4·3	5·2	6·2	8·0	9·9	10·8	11·7	4·6	5·4	6·4	8·2	10·0	10·9	11·8
33–45 mo	4·3	5·1	6·1	7·9	9·7	10·7	11·6	4·5	5·4	6·3	8·1	9·8	10·8	11·6
34–46 mo	4·2	5·1	6·0	7·8	9·6	10·6	11·5	4·5	5·3	6·2	8·0	9·7	10·6	11·4
35–47 mo	4·2	5·0	6·0	7·7	9·5	10·5	11·3	4·4	5·2	6·1	7·9	9·6	10·5	11·3
36–48 mo	4·1	5·0	5·9	7·7	9·4	10·4	11·2	4·4	5·2	6·1	7·8	9·5	10·3	11·2
37–49 mo	4·1	4·9	5·9	7·6	9·3	10·2	11·1	4·3	5·1	6·0	7·7	9·3	10·2	11·0
38–50 mo	4·1	4·9	5·8	7·5	9·2	10·1	11·0	4·3	5·1	6·0	7·6	9·3	10·1	10·9
39–51 mo	4·1	4·9	5·8	7·5	9·2	10·0	10·9	4·3	5·0	5·9	7·5	9·2	10·0	10·8
39–51 mo	4·1	4·9	5·8	7·5	9·2	10·0	10·9	4·3	5·0	5·9	7·5	9·2	10·0	10·8
40–52 mo	4·0	4·9	5·7	7·4	9·1	10·0	10·8	4·2	5·0	5·9	7·5	9·1	9·9	10·7
41–53 mo	4·0	4·8	5·7	7·3	9·0	9·9	10·7	4·2	5·0	5·8	7·4	9·0	9·8	10·6
42–54 mo	4·0	4·8	5·7	7·3	8·9	9·8	10·6	4·2	4·9	5·8	7·3	8·9	9·7	10·5
43–55 mo	4·0	4·8	5·6	7·2	8·8	9·7	10·5	4·1	4·9	5·7	7·3	8·8	9·7	10·4
44–56 mo	4·0	4·8	5·6	7·2	8·8	9·6	10·4	4·1	4·9	5·7	7·2	8·8	9·6	10·3
45–57 mo	4·0	4·7	5·6	7·1	8·7	9·5	10·3	4·1	4·8	5·6	7·2	8·7	9·5	10·2
46–58 mo	4·0	4·7	5·5	7·1	8·6	9·5	10·2	4·1	4·8	5·6	7·1	8·6	9·4	10·2
47–59 mo	4·0	4·7	5·5	7·0	8·6	9·4	10·1	4·1	4·8	5·6	7·1	8·6	9·4	10·1
48–60 mo	4·0	4·7	5·5	7·0	8·5	9·3	10·0	4·0	4·8	5·5	7·0	8·5	9·3	10·0
49–61 mo	3·9	4·7	5·5	7·0	8·4	9·2	10·0	4·0	4·7	5·5	7·0	8·4	9·2	9·9
50–62 mo	3·9	4·7	5·4	6·9	8·4	9·2	9·9	4·0	4·7	5·5	6·9	8·4	9·2	9·9
51–63 mo	3·9	4·6	5·4	6·9	8·3	9·1	9·8	4·0	4·7	5·4	6·9	8·3	9·1	9·8
52–64 mo	3·9	4·6	5·4	6·8	8·3	9·0	9·7	4·0	4·7	5·4	6·8	8·3	9·0	9·7
53–65 mo	3·9	4·6	5·4	6·8	8·2	9·0	9·6	3·9	4·6	5·4	6·8	8·2	9·0	9·7
54–66 mo	3·9	4·6	5·3	6·7	8·1	8·9	9·6	3·9	4·6	5·3	6·8	8·2	8·9	9·6
55–67 mo	3·9	4·6	5·3	6·7	8·1	8·8	9·5	3·9	4·6	5·3	6·7	8·1	8·8	9·5
56–68 mo	3·9	4·6	5·3	6·7	8·0	8·7	9·4	3·9	4·6	5·3	6·7	8·0	8·8	9·4
57–69 mo	3·9	4·6	5·3	6·6	8·0	8·7	9·3	3·9	4·5	5·3	6·6	8·0	8·7	9·4
58–70 mo	3·9	4·6	5·3	6·6	7·9	8·6	9·2	3·9	4·5	5·2	6·6	7·9	8·7	9·3
59–71 mo	3·9	4·6	5·2	6·5	7·8	8·5	9·2	3·8	4·5	5·2	6·5	7·9	8·6	9·2

Mo, months; SND, standard normal deviate.

* 0–23-month measurement reflects recumbent length and 24–72 months reflect standing height.

† Age (months = mo) at the start of interval.

‡ SND is the standard normal deviate also defined as 1 *Z*-score.

§ Modeled median.

**Fig. 4:**
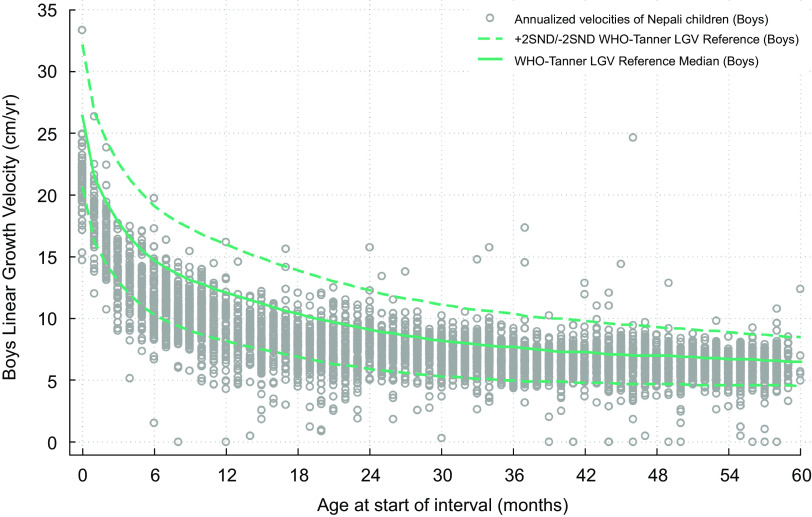
Annualised linear growth velocities of Nepali boys aged 0–59 months at the start of the growth interval, in the *Tarai* measured between 2013 and 2016, plotted against the WHO-Tanner modelled linear growth velocity reference curve (median + 2 *Z*-score)

**Fig. 5:**
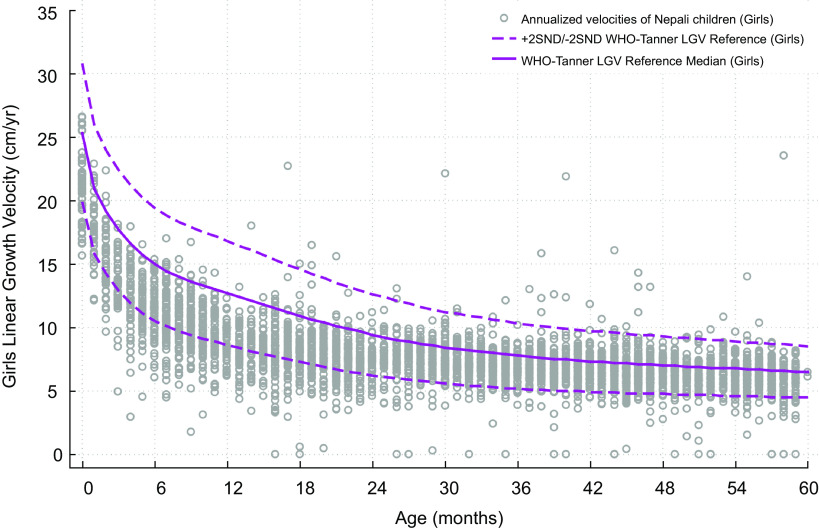
Annualised linear growth velocities of Nepali girls aged 0–59 months at the start of the growth interval, in the *Tarai* measured between 2013 and 2016, plotted against the WHO-Tanner modelled linear growth velocity reference curve (median + 2 *Z*-score)

Figures 4 and 5 reflect on the performance of the modelled WHO-Tanner reference by plotting annualised LGV of the population sample of Nepali children across all preschool ages against the sex-specific curves. In this study population sample of 4276 children, 53·4 % were boys and 46·6 % girls (data not shown), both for whom ∼83 % of growth velocities were below the reference median across all ages. LGVZ distributions and percentages of faltering velocities (<-2 LGVZ) by sex within age strata are summarised in Table 2, revealing at each age group < 24 months (<6, 6–11 and 12–23 months) mean (sd) age-specific LGVZ of −1·4 (1) and −1·6 (1) for boys and girls, accompanied by growth faltering velocities of ∼25 % among boys and ∼35 % among girls. Markedly improved growth rates relative to the reference are evident starting in the 3^rd^ year, evident by a mean (sd) annual LGVZ of −0·7 (0·9) and −0·8 (1) that plateaus each year at −0·6 (∼1) and −0·5 (∼ −1·5) thereafter in boys and girls, respectively. Approximately 6∼7 % of children of both sexes each year starting from 24 to 59 months experience growth faltering. Normalised growth velocities, expressed as mean LGVZ and prevalence of growth faltering, expressed as % LGVZ < -2, in relation to the sex-age specific reference, were lower for girls than boys at each age group < 48 months (*P*-value <0·05) (Table 2).

**Table 2:** Annualised linear growth velocities and prevalence of growth faltering by sex and age at start of interval among Nepali pre-school aged children

Age (mo)	Total (*n* 4276)				Boys (*n* 2269)					Girls (*n* 2007)				
	LGVZ		LGVZ < -2		Age (mo)	LGVZ		LGVZ < -2			LGVZ		LGVZ < -2	
	Mean	sd	%	95 % CI*		Mean	sd	%	95 % CI*	Age (mo)	Mean	sd	%	95 % CI*
< 6 (*n* 636)	−1·5	1·0	31·1	26·8, 35·8	< 6 (*n* 344)	−1·4^†^	1·0	26·5^§^	20·9,32·9	< 6 (*n* 292)	−1·6^‡^	1·0	36·6^§^	30·9, 42·8
6–11·9 (*n* 976)	−1·5	0·9	28·6	23·3,34·5	6–11·9 (*n* 519)	−1·4^‡^	0·9	24·3^§^	19·8, 29·4	6–11·9 (*n* 457)	−1·6^‡^	0·9	33·6^§^	26·4, 41·5
12–23·9 (*n* 717)	−1·5	1·0	29·3	25·0, 34·1	12–23·9 (*n* 392)	−1·4^‡^	1·0	25·4^§^	20·5, 31·0	12–23·9 (*n* 325)	−1·6^‡^	1·0	34·1^§^	29·8, 38·6
24- 35·9 (*n* 654)	−0·8	0·9	6·7	5·5, 8·2	24- 35·9 (*n* 340)	−0·7	0·9	6·3	5·0, 7·9	24- 35·9 (*n* 314)	−0·8	1·0	7·2	5·5, 9·3
36–47·9 (*n* 673)	−0·5	1·1	6·2	5·0, 7·6	36–47·9 (*n* 351)	−0·6^‡^	1·1	6·4	4·9, 8·2	36–47·9 (*n* 322)	−0·5^‡^	1·2	6·0	4·3, 8·3
48–59·9 (*n* 620)	−0·5	1·1	6·2	4·6, 8·2	48–59·9 (*n* 323)	−0·6	1·0	6·2	4·6, 8·4	48–59·9 (*n* 297)	−0·5	1·1	6·1	4·4, 8·5

Mo, months; LGVZ, linear growth velocity *Z*-score.

* 95 % CI adjusted for clustering using robust estimation of standard errors.

† *P* < 0·05; *t*-test conducted within each age strata.

‡ *P* < 0·05; Pearson’s chi-square conducted within each age strata.

§ Linear growth velocity *Z*-score (LGVZ).

We explored whether growth faltering children differed by initial L/HAZ (Table 3). Among infants <6 months, rates of subsequent faltering were twice as high among those with an initial LAZ > -2 (32·4 %) than those whose initial LAZ was <-2 (17·9 %) (*P* < 0·05). Thereafter, the proportion of children experiencing growth faltering was comparable among stunted and not stunted children whose intervals started at 6–11·9 and 12–23·9 months of age, averaging 28·6 % and 29·3 %, respectively, revealing a population of young preschoolers growing poorly. Among older children (>24 months), proportion exhibiting growth velocity faltering was ∼6 % regardless of initial HAZ.

**Table 3:** Proportion of Nepali children with linear growth faltering* by height for age (L/HAZ) status and age stratum at the start of an interval

	0–5·9 mo		6–11·9 mo		12–23·9 mo		24–35·9 mo		36–47·9 mo		48–59·9 mo	
Age	LGVZ < -2											
Initial L/HAZ	%	*n* ‡	%	*n* ‡	%	*n* ‡	%	*n* ‡	%	*n* ‡	%	*n* ‡
>-2	32·4	580§	28·9	810	28·0	1020	7·1	889	6·8	990	6·5	1018
<-2†	17·9	56§	27·3	172	31·4	645	6·2	755	5·4	757	5·7	633
	%	*n* ||	%	*n* ||	%	*n* ||	%	*n* ||	%	*n* ||	%	*n* ||
Total	31·1	636	28·6	982	29·3	1665	6·7	1644	6·2	1747	6·2	1652

Mo: months; LGVZ: linear growth velocity *Z*-score; HAZ: height-for-age *Z*-scores.

* Linear growth faltering: LGVZ < -2.

† L/HAZ < -2: stunting.

‡ ‘*n*’ reflects the row total for children within each age interval.

§ *P* < 0·05, chi-square test.

|| ‘*n*’ reflects the column total for children within each age interval.

## Discussion

Periodic anthropometric surveys remain the mainstay of population assessment and monitoring progress towards eliminating preschool growth failure as a public health problem. Surveys document the prevalence and risk factors of stunting; however, they are (a) unable to pinpoint the timing or reveal extent or severity of growth faltering, (b) likely to misclassify the comparison group by including an unknown proportion of children not classified as stunted whose growth may be in decline and thus unable to (c) identify population groups of non-stunted children growing slowly and at risk of becoming stunted or (d) potentially modifiable risk factors that could attenuate growth faltering.

Building off conducted surveys, we propose that reassessment of a proportion preschool-aged children a year later provides the potential to establish distributions of annual growth velocities. With an interpretable LGV reference, it is possible to estimate the prevalence of growth faltering by sex and identify groups of non-stunted children at greatest risk of growth faltering by location, socio-economic, nutritional, morbidity and other assessed characteristics. Growth velocity at 12-month intervals can also be utilised to understand trends in growth faltering patterns over time that can be more revealing of children’s health and diet than is a static measure of nutritional status, which reflects the accumulated effects of nutrition, health and genetic influences on growth^(41)^. A 12-month interval presents advantages over shorter intervals in that it represents the summed effects of all seasons of the year on child growth, being more agnostic to calendar month of initial and follow-up assessments and thus more interpretable and potentially comparable across populations.

To our knowledge, no single, annual LGV reference exists for use across the entire preschool age range. Among existing references, growth rates have been reported for intervals shorter than a year and are not calibrated by each month of age. A *de novo* multi-country, annual growth velocity reference derived from growth data of healthy children growing in supportive environments is acknowledged to be the gold standard metric. But currently, it does not exist and carries a high cost burden and will take many years to plan, execute, analyse and publish^(42)^. The approach detailed in this article presents a pragmatic referent tool for use in the medium term with velocity distributions concatenated from sequentially ageing, generally healthy children from well-published studies to generate plausible, sex-specific LGV curves that are continuous throughout the preschool years. The resulting growth velocity patterns reveal continuous, plausible, age-specific rates of growth among healthy children manifest by early, rapid curvilinear deceleration from a peak after birth through the 2^nd^ year, followed by a far shallower decline^(43)^, consistent with what attained growth charts depict^(15,40)^. To guide use and interpretation of the reference, we propose that annual LGV below −2 *Z*-scores be classified as growth faltering, analogous to the conventional use of *Z*-scores in attained anthropometric assessment.

When applied and interpreted against a multi-year cohort study of children representative of Nepal’s *Tarai,* findings revealed that ∼30 % of children <24 months of age and ∼7 % of older preschoolers were faltering, consistent with trends in stunting gleaned from static assessments in Nepal^(44)^ and region^(45)^ and longitudinal preschoolers studies in low-middle income countries. Unique to this approach is an ability to reveal the timing and extent of growth faltering among groups of children by their initial nutritional status and many other risk factors that can be initially assessed in a survey and reassessed at follow-up. The approach provides the basis for characterising growth and targeting for monitoring groups of children at high risk at the population-level, along with possibility of fashioning future interventions. For example, in the Nepal study, risk of a subsequent low growth velocity concentrated in girls at most age groups, children with a low weight for height and those whose mother was short in stature and household scored low on a locally constructed wealth index^(46)^.

In Nepal, approximately 30 % of already stunted children <24 months at the outset continued to falter in linear growth, the exception being stunted infants <6 months, among whom the percentage was 17 %, possibly reflecting disproportionate recovery, or ‘catch-up’ linear growth if born small-for-gestational age^(47,48)^. However, a similar percentage of children <24 months who were above −2 L/HAZ at the outset (∼30 %) also faltered during the following year. Such children are undetectable in cross-sectional surveys, (mis)classified as normal, yet represent a potential segment of the population to target for prevention to reduce the prevalence of stunting in a population. This is consistent with findings from other studies where it has been revealed that solely relying on HAZ misinterprets the extent of accumulated growth deficits in resource-constrained populations^(49)^. Among older preschoolers, comparable percentages of stunted and non-stunted (∼6 %) also exhibited abnormally low growth velocities.

Among the referent’s limitations are its unorthodox construction, which include choice of growth reference studies, the span in decades in which they were conducted and methods employed to annualise growth rates and derive standard deviations that are being interpreted as normal deviates. The Tanner reference was derived from a relatively small sample of children living in Central London decades ago and cannot be taken to be represent multiregional settings like the WHO Child Growth Standard is nor can it be considered prescriptive as a reference and based on its location and sampling characteristics. To this point, underlying our approach is a substantial evidence of children achieving comparable growth rates in supportive environments^(15,20,24,50)^, with which the Tanner and Zurich (see online Supplemental Tables 3(a) and (b)) cohort comparison was consistent. Another unorthodoxy lies with converting the 6-monthly WHO growth increments to annual estimates by assuming their additivity at each initial month of age. Additionally, we could not standardise ages of assessment across the studies used to create the WHO-Tanner reference, both of which used slightly different age intervals – WHO used full year of attained age intervals and Tanner used the midpoint of the age interval to estimate velocity. These are limitations that would be adequately addressed in designing and executing a rigorous, multi-country, longitudinal study designed to generate prescriptive annual velocity distributions by month of age and sex, though such a global reference can be expected to take a decade or longer to plan, fund, execute, analyse and publish. To our knowledge, neither of the studies on which the novel metric is based, collected gestational age at birth data, which reflects that a factor adjustment may be appropriate when defining a postnatal growth velocity reference. However, most current in-country cohorts in low-middle income countries to which this reference would be applied also lack gestational age data. Finally, we acknowledge the potential for error when subtracting recumbent length from standing height. Standing height in 2-year-old children is approximately 0·7 cm less than recumbent length^(51)^ which, in this reference, could bias the computed annualised velocity estimate for children that are transitioning from 2 to 3 years of age during the study period. This might be expected to lead to incongruous joints when combining curves, which was not observed.

In conclusion, assessing LGV in child populations remains an under-utilised approach to reveal the extent, timing, duration and groups at highest risk of abnormal growth deceleration – a process that necessarily precedes postnatal stunting. Detailed assessment of socio-economic status, food security, morbidity, diet and other biomarkers assessed at the outset (and end) of an interval can lead to characterising, at a population level, groups of children reliably at high risk of growth faltering, irrespective of initial height-for-age, who could benefit from health and nutrition services seeking to preserve normal growth in different low-middle income country settings, which offers an area for future research. We propose that measurement of annualised growth velocities coupled with use of this reference and in-depth epidemiological risk factor assessments may assist countries in pursuit of their Sustainable Development Goal targets and open new research and programme approaches to preventing stunting.

## Supporting information

Manohar et al. supplementary material 1 Manohar et al. supplementary material

Manohar et al. supplementary material 2 Manohar et al. supplementary material

Manohar et al. supplementary material 3 Manohar et al. supplementary material

Manohar et al. supplementary material 4 Manohar et al. supplementary material

Manohar et al. supplementary material 5 Manohar et al. supplementary material

## Supplementary material

For supplementary material accompanying this paper visit https://doi.org/10.1017/S1368980023002409
