# Supplementary material for: Evaluating preschool linear growth velocities: an interim reference illustrated in Nepal
Source: Public Health Nutr. 2023 Nov 7;26(12):2704–16. doi: 10.1017/S1368980023002409 (PMC10755438; doi:10.1017/S1368980023002409)
Supplement: Manohar et al. supplementary material 1 — Manohar et al. supplementary material [file S1368980023002409sup001.docx]

**Evaluating preschool linear growth velocities:  An interim reference illustrated in Nepal**

Swetha Manohar, Elizabeth Colantuoni, Andrew L. Thorne-Lyman, Binod Shrestha, Ramesh Kant Adhikari, Angela KC, Abhigyna Bhattarai, Keith P. West Jr.

**Online Supporting Material**

**Table of Contents**

1. **Section I:** Review of existing velocity references.
2. **Supplemental Table 1:** Review of longitudinal studies reporting linear growth velocities considered for annualized reference.
3. **Supplemental Table 2:** Formulae used to estimate annualized linear growth velocities.
4. Section II: Basis for generating a twelve-month linear growth reference and details of the WHO Child Growth Standard and Tanner Reference populations.
5. **Supplemental Table 3A**: Comparison of variation in 12-month velocity for Tanner Reference & Zurich Longitudinal Study of Growth and Development Reference (girls).
6. **Supplemental Table 3B**: Comparison of variation in 12-month velocity for Tanner Reference & Zurich Longitudinal Study of Growth and Development Reference (boys).
7. **Section III:** References for Online Supporting Material
8. **Supplemental Table 4:** Derived 12-month increments (unmodeled) estimates for length/height of the WHO- Tanner linear growth velocity reference.
9. Supplemental Figure 1: WHO-Tanner sex-combined derived referent median scatterplot and modeled 12-month linear velocities through 59 months of age.
10. Supplemental Figure 2: WHO-Tanner modeled sex-combined linear growth velocity reference curve through 59 months of age (median + 2 standard normal deviate or z-score).
11. Supplemental Figure 3: WHO-Tanner sex-specific derived variance in linear growth velocity scatterplot and modeled variance in linear growth velocity through 59 months of age.
12. Supplemental Figure 4: Study Flow diagram for inclusion into the analytic sample for 0-59 month old Nepali children from the Tarai from whom measurements were taken between 2013- 2016
13. **Section I:** Review of existing velocity references

We first reviewed the literature to identify if any references existed that could serve as a growth reference with annualized growth increments for each month of age initiating at ages: 0 (< 1 month) to 59 months age. The criteria included: (1) reported 12-month length/height (hereafter referred to as height) increments, with a preference for the latter; (2) included corresponding measures of statistical uncertainty (i.e., standard deviations) for velocity estimates; (3) were available by month for ages starting from 0 to 59 months; (4) were sex-specific and (5) were conducted in seemingly supportive dietary/nutritional, health care, family and environmental conditions. No single reference candidate was identified. We then broadened criteria (1) to include references that reported 3-month, 6-month or 12-month length/height (hereafter referred to as height) increments, with a preference for the latter. Several studies partially met our criteria, including the Fels Longitudinal Study, carried out in Ohio, USA from 1929-1978 ^(1–3)^, which provided 3-month to 6-month linear growth estimates (depending on age) which, in part, served as a basis for the CDC/NCHS 2000 growth velocity references ^(4)^. Additional studies included in our review were Prader and colleagues’ Zurich Longitudinal Study, conducted between 1954 and 1976, which measured velocities at varying intervals among Swiss children from birth to 18 years ^(5)^, annual growth charts published by Tanner and colleagues from the United Kingdom in 1966 ^(6)^; 3-month growth increment data collected among infants in Idaho, USA by Fomon and colleagues published in 1991^(7)^, and the 2006 WHO Multicentre Growth Reference Study (MGRS) Child Growth Standards, with 6-month increments through an interval-ending age of 24 months, based on studies conducted in multiple countries ^(8)^. We present details of these reference – their study population, measurements and age range covered. Ultimately, the references that best suit our purpose and offered the most complete data to construct an annualized growth velocity curve through birth to preschool age (<59 months) were WHO Growth Standard 6-month velocity reference and the Tanner Height Velocity reference.

1. **Supplemental Table 1:** Review of longitudinal studies reporting linear growth velocities considered for annualized reference.

| Study/Reference Name | Fels Longitudinal Study Collection^[[1]](#footnote-1),^^[[2]](#footnote-2)^ | Iowa Study & Fels Study Reference^[[3]](#footnote-3)^ | Tanner Velocity Standards from Birth to Maturity^[[4]](#footnote-4)^ | First Zurich Longitudinal Study of Growth and Development^[[5]](#footnote-5)^ | WHO Growth Velocity Standards^[[6]](#footnote-6)^ |
| --- | --- | --- | --- | --- | --- |
| Study years | 1929 - 1978 | 1965 – 1989  1929 – 1978 | 1946 - 1954 | 1954- 1976 | 1997 - 2003 |
| Age range | 0-18 y | 0-2 y | 0-12 y | 2-18 y | 0-2 y |
| Increment range for velocity estimates | 3 mo; ages 0-1 y  6 mo; ages 1-18 y | Daily; 0-6 months  3 mo; 0-24 months | 3 mo; ages 0-2 y  12 mo; ages 2-6 y | 3 mo; ages 0-1 y  6 mo; ages 1-2 y  12 mo; ages 2- 18 y | 1, 3, 6 mo |
| Breastfeeding (BF) status | Non-BF, predominantly  formula-fed | Non-exclusively BF and formula fed | Non-exclusively BF and formula fed | Not stated | Exclusively BF |
| Sample size | N=495  (256 girls, 239 boys) | N=1142: 0-3 mo  (BF: 200 boys, 214 girls, formula fed: 380 boys, 384 girls)  N=496: 3-6 mo  357: 6-24 mo | N=160  (80 girls, 80 boys) | N=413  (207 girls, 206 boys) | N=882  (454 girls, 428 boys) |

**Supplemental Table 1 contd.:** Review of longitudinal studies reporting linear growth velocities considered for annualized reference.

| Study/Reference Name | Fels Longitudinal Study Collection^[[7]](#footnote-7)^ | Iowa Study & Fels Study Reference^[[8]](#footnote-8)^ | Tanner Velocity Standards from Birth to Maturity^[[9]](#footnote-9)^ | First Zurich Longitudinal Study of Growth and Development^[[10]](#footnote-10)^ | WHO Growth Velocity Standards^[[11]](#footnote-11)^ |
| --- | --- | --- | --- | --- | --- |
| Anthropometry  method | Recumbent length:  < 2 y | Recumbent length:  < 2 y | Supine lengths:  1 mo – 2 y  Standing height:  > 2 y | Supine length:  0-14 y  Standing height:  2-19 y | Recumbent length:  < 2 y |
| Location | Ohio - United States | Iowa - United States  Ohio- United States | London -United Kingdom | Zurich, Switzerland | Muscat -Oman  Accra-Ghana  Oslo – Norway  Pelotas- Brazil  South Delhi – India  California- United States |

1. **Supplemental Table 2:** Formulae used to estimate annualized linear growth velocities.

| Measurement/ Metric | Formula | Data Source |
| --- | --- | --- |
| Annualized LV | For n{0,12},  Median _LVn_ = WHOLV _(n…n+5*)_  WHOLV_(n+5---n+11_^†^_)_  where ‘n’ is the age in months in the set 0 to 12 and n=0 is the sum of the WHOLV (from 0 through 5 months of age) plus WHO (for 6 through 11 months of age) | WHO Length Velocity Standards |
| Annualized HV | $\surd$(HV for preceding age interval^2^ + HV for succeeding age interval^2^)    2 | Tanner Height Velocity Reference |
| SD _(Annualized LV)_ | SD _(Annualized LGV)_ = {Median - (-1SD) + (1SD - Median)}  2 | WHO Length Velocity Standards |
| SD _(Annualized HV)_ | SD _(Annualized LGV)_ = {Median - (-2SD)}  2 | Tanner Height Velocity Reference |
| Δ Height _(Age_ti,i)_ | (Height _(t+1,i)_ - Height _ti_ ) x 365.25  # Days between consecutive measurements  where ‘i’ is the subject and ‘t’ is the assessment number and ‘age_ti’ is the age of child ‘i’ at assessment | Nepal PoSHAN Community Studies Data |
| LGVZ | Annualized height^††^velocity _(PoSHAN)_ – median height^††^velocity _(WHO-Tanner Velocity Reference)_  SD for median height velocity _(WHO-Tanner Derived Reference)_ | Nepal PoSHAN Community Studies & WHO-Tanner Velocity Reference  Curve |

LV= Length velocity, directly estimates using indicated growth standard

WHOLV = WHO Child Growth Standard Length Velocity

HV = Height velocity, direct estimates using indicated growth reference

LGVZ= Linear growth velocity z-score, estimated using modeled regression estimates from the derived annualized growth reference.

SD= Standard Deviation

*WHO Child Growth Standard label this age interval as ‘0-6’ months

^†^ WHO Child Growth Standard label this age interval as ‘6-12’ months

^††^ Height or length if < 2 years

1. **Section II:** Basis for generating a twelve-month linear growth reference and details of the

WHO Child Growth Standard and Tanner Reference populations

The WHO Child Growth Standard data was collected from children sampled to participate in a longitudinal component of population based study, MGRS, from Brazil, Ghana, India, USA, Oman and Norway (n=882) ^(9)^. Data was collected from 1997 to 2003. The children came from unconstrained socio-economic backgrounds, whose mothers were breastfeeding, introducing complementary foods in a timely and appropriate way. These data provided 6-month velocity intervals for every month of age from 0-24 month providing us a structure for which no imputations were required to assemble annualized growth velocities using 6-month velocity estimates from 0-12 months using the ‘annualized LV’ formula in **Supplemental Table 2**.

The Tanner reference was developed from a sample of children (n=160) in Central London who were randomly selected from records of families having regular health checks at the University of London’s Institute of Child Health and belonged to the Child Study Center group from 1952 to 1954, who were assumed to represent urban British children ^(6)^. The resultant curves from this study have been widely applied over the decades as a reference for assessing child growth velocity^(6,10–13)^. The Tanner reference provided annualized velocity intervals every 3 months from 12 months of age and every 6 months from 33 months of age onwards. This necessitated interpolation of median and standard deviations using the ‘annualized HV’ formula (**Supplemental Table 2)** to compute 12-month velocities for months of age that did not have a reported annual velocity**.** The growth intervals presented in the Tanner reference we close enough (3-6 months) to allow for this interpolation. The complete combined twelve-month linear growth velocity reference in tabular format prior to curve constructions can be reviewed in **Supplemental Table 4.**

We accepted that these datasets fairly represent child growth velocity distributions, a condition that has been shown to support unconstrained and comparable growth regardless of geographic location and genotype ^(14–18)^. This understanding is supported by the WHO Child Growth Standards’ MGRS data which show little variability in average growth by age in children across its five study countries ^(18)^. Further, while it was not possible to compare the Tanner reference to many other references given there were only a minimal number of twelve-month growth reference, we compared the Tanner reference population to the Zurich Longitudinal Study of Growth and Development population for boys and girls, to the extent possible given each reference reports 12-month median velocities at slightly different ages (**Supplemental Tables 3A & 3B**). The tables present 12-month median velocities and respective variation (SD) for a sample age range that reflects the age range captured by the combined annual growth velocity reference presented in this paper informed by the Tanner reference. With the exception of the 15/16-month age mark, the variation around the medians for the Tanner and Zurich references are comparable. Further, the Zurich reference provides limited comparable, reliable data for the intervals starting at 21 months for girls and boys (n=5, n=11 respectively) and at 30 months for boys (n=16). Differences observed at those age intervals can be attributed to sampling error in the Zurich study given small sample sizes and given highly comparable estimates at other age-sex intervals with larger samples. Finally, on combining the WHO-Tanner reference, we observed that medians and SDs in linear growth velocity for both populations aligned without apparent discontinuity, with respect to their junctures and slopes (**Supplemental Figure 1**).

1. **Supplemental Table 3A**: Comparison of variation in 12-month velocity for Tanner Reference & Zurich Longitudinal Study of Growth and Development Reference (girls)

|  | **Tanner Reference (girls)** | | | | **Zurich Reference (girls)** | | | | |
| --- | --- | --- | --- | --- | --- | --- | --- | --- | --- |
| **Age (year)** | **Age (months)*** | **N** | **p50**  **(median)** | **SD** | **Age (years)** | **Age (months)*** | **N** | **p50**  **(median)** | **SD** |
| 1.37 | 16.4 | ~160 | 11.8 | **1.9** | 1.25 | 15.0 | 54 | 10.1 | **2.7** |
| 1.87 | 22.4 | ~160 | 9.6 | **1.7** | 1.75 | 21.0 | 5 | 10.1 | **4.3** |
| 2.75 | 33.0 | ~160 | 8.1 | **1.4** | 2.50 | 30.0 | 129 | 9.4 | **1.2** |
| 3.75 | 45.0 | ~160 | 7.2 | **1.2** | 3.50 | 42.0 | 128 | 7.5 | **1.0** |
| 4.75 | 57.0 | ~160 | 6.6 | **1.1** | 4.50 | 54.0 | 127 | 6.6 | **1.1** |
| 5.75 | 69.0 | ~160 | 6.2 | **1.0** | 5.50 | 66.0 | 135 | 6.2 | **0.9** |
| * Age in months approximated based on age in years reported by each reference; reported age and related annualized velocities do not align between references   1. **Supplemental Table 3B**: Comparison of variation in 12-month velocity for Tanner Reference & Zurich Longitudinal Study of Growth and Development Reference (boys) | | | | | | | | | |
|  | **Tanner Reference (boys)** | | | | **Zurich Reference (boys)** | | | | |
| **Age (year)** | **Age (months)*** | **N** | **p50**  **(median)** | **SD** | **Age (years)** | **Age (months)*** | **N** | **p50**  **(median)** | **SD** |
| 1.37 | 16.4 | ~160 | 11.1 | **1.9** | 1.25 | 15.0 | 52 | 11.7 | **2.5** |
| 1.87 | 22.4 | ~160 | 9.2 | **1.7** | 1.75 | 21.0 | 11 | 10.0 | **2.2** |
| 2.75 | 33.0 | ~160 | 8.0 | **1.3** | 2.50 | 30.0 | 16 | 9.8 | **1.0** |
| 3.75 | 45.0 | ~160 | 7.2 | **1.2** | 3.50 | 42.0 | 43 | 7.8 | **1.0** |
| 4.75 | 57.0 | ~160 | 6.6 | **1.1** | 4.50 | 54.0 | 91 | 6.7 | **0.8** |
| 5.75 | 69.0 | ~160 | 6.2 | **1.0** | 5.50 | 66.0 | 128 | 6.3 | **0.9** |

* Age in months approximated based on age in years reported by each reference; reported age and related annualized velocities do not align between references

1. **Section III: References for Online Supporting Material**

1. Baumgartner RN, Roche AF & Himes JH (1986) Incremental growth tables: Supplementary to previously published charts. Am. J. Clin. Nutr. **43**, 711–722.

2. Guo S, Roche AF, Fomon SJ, et al. (1991) Reference data on gains in weight and length during the first two years of life. J. Pediatr. **119**, 355–362.

3. Roche AF, Guo S & Moore WM (1989) Weight and recumbent length from 1 to 12 mo of age: Reference data for 1-mo increments. Am. J. Clin. Nutr. **49**, 599–607.

4. Kuczmarski RJ, Ogden CL, Grummer-Strawn LM, et al. (2000) CDC growth charts: United States. Adv. Data.

5. Prader A, Largo RH, Molinari L, et al. (1989) Physical growth of Swiss children from birth to 20 years of age. First Zurich longitudinal study of growth and development. Helv Paediatr Acta Suppl. **52**, 1–125.

6. Tanner JM, Whitehouse RH & Takaishi M (1966) Standards from birth to maturity for height, weight, height velocity, and weight velocity: British children, 1965 Part II. Arch. Dis. Child. **41**, 613–635.

7. Nelson SE, Rogers RR, Ziegler EE, et al. (1989) Gain in weight and length during early infancy. Early Hum. Dev. **19**, 223–239.

8. WHO Multicentre Growth Reference Study Group (2009) WHO Child Growth Standards: Growth velocity based on weight, length and head circumference. Methods and development. Geneva.

9. de Onis M, Garza C, Victora CG, et al. (2004) The WHO Multicentre Growth Reference Study: Planning, study design, and methodology. Food Nutr. Bull. **25**, S15-26.

10. Cole TJ (2012) The development of growth references and growth charts. Ann. Hum. Biol. **39**, 382–394.

11. Bozzola M & Meazza C (2011) Growth Velocity Curves: What They Are and How to Use Them. In Handb. Growth Growth Monit. Heal. Dis. Pavia: .

12. Kolsteren PW, Kusin JA & Kardjati S (1997) Pattern of linear growth velocities of infants from birth to 12 months in Madura, Indonesia. Trop. Med. Int. Heal. **2**, 291–301.

13. Schwinger C, Fadnes LT & Broeck J Van Den (2016) Using growth velocity to predict child mortality 1 , 2. Am. J. Clin. Nutr. **103**, 801–807.

14. Spencer PR, Sanders KA & Judge DS (2018) Growth curves and the international standard: How children’s growth reflects challenging conditions in rural Timor-Leste. Am. J. Phys. Anthropol. **165**, 286–298.

15. Habicht JP, Yarbrough C, Martorell R, et al. (1974) Height and Weight Standards for Preschool Children. How Relevant Are Ethnic Differences in Growth Potential ? Lancet **303**, 611–615.

16. Martorell R (1985) Child growth retardation: a discussion of its causes and its relationship to health. In Nutr. Adapt. Man.

17. Martorell R (2010) Physical growth and development of the malnourished child: Contributions from 50 years of research at INCAP. Food Nutr. Bull. **31**, 68–82.

18. Victora CG, De Onis M, Garza C, et al. (2000) Growth patterns of breastfed infants in seven countries. Acta Paediatr. Int. J. Paediatr. **89**, 215–222.

1. Supplemental Table 4: Derived 12-month increments (unmodeled) estimates for length/height of the WHO- Tanner linear growth velocity reference.

| **Age Interval** | **-3 SND**^†^ | **-2 SND**^†^ | **-1 SND**^†^ | **Median** | **3 SND**^†^ | **2 SND**^†^ | **1 SND**^†^ | **SND**^†^ **(Median)** | **CV**^‡^ |
| --- | --- | --- | --- | --- | --- | --- | --- | --- | --- |
| **0-12 mo** | 16.8 | 19.3 | 22.0 | 24.7 | 27.5 | 30.4 | 33.5 | 2.8 | 11.1 |
| **1-13 mo** | 14.3 | 16.7 | 19.2 | 21.8 | 24.4 | 27.3 | 30.1 | 2.6 | 11.9 |
| **2-14 mo** | 12.2 | 14.5 | 16.9 | 19.4 | 21.9 | 24.6 | 27.4 | 2.5 | 12.9 |
| **3-15 mo** | 10.9 | 13.0 | 15.3 | 17.6 | 20.2 | 22.7 | 25.4 | 2.5 | 13.9 |
| **4-16 mo** | 9.8 | 12.0 | 14.1 | 16.4 | 18.8 | 21.3 | 23.9 | 2.4 | 14.3 |
| **5-17 mo** | 9.1 | 11.2 | 13.3 | 15.6 | 17.9 | 20.4 | 22.9 | 2.3 | 14.7 |
| **6-18 mo** | 8.7 | 10.6 | 12.7 | 14.9 | 17.2 | 19.6 | 22.1 | 2.3 | 15.1 |
| **7-19 mo** | 8.2 | 10.1 | 12.2 | 14.4 | 16.6 | 19.1 | 21.5 | 2.2 | 15.3 |
| **8-20 mo** | 7.8 | 9.7 | 11.8 | 13.9 | 16.1 | 18.5 | 21.0 | 2.2 | 15.5 |
| **9-21 mo** | 7.4 | 9.3 | 11.3 | 13.4 | 15.7 | 18.0 | 20.5 | 2.2 | 16.4 |
| **10-22 mo** | 7.1 | 9.0 | 11.0 | 13.1 | 15.3 | 17.6 | 20.0 | 2.2 | 16.4 |
| **11-23 mo** | 6.8 | 8.6 | 10.6 | 12.7 | 14.9 | 17.2 | 19.5 | 2.2 | 16.9 |
| **12- 24 mo** | 6.5 | 8.3 | 10.2 | 12.3 | 14.5 | 16.8 | 19.1 | 2.2 | 17.5 |
| **13-25 mo** | * | 9.7 | 10.9 | 13.5 | 14.9 | 16.1 | 17.3 | 2.0 | 15.0 |
| **14-26 mo** | * | 9.0 | 10.2 | 12.7 | 14.0 | 15.2 | 16.4 | 1.9 | 14.7 |
| **15-27 mo** | * | 9.0 | 10.2 | 12.7 | 14.0 | 15.2 | 16.4 | 1.9 | 14.7 |
| **16-28 mo** | * | 8.2 | 9.3 | 11.8 | 13.1 | 14.3 | 15.5 | 1.9 | 16.4 |
| **17-29 mo** | * | 8.0 | 8.8 | 11.2 | 12.5 | 13.6 | 14.7 | 1.8 | 15.7 |
| **18-30 mo** | * | 8.0 | 8.8 | 11.2 | 12.5 | 13.6 | 14.7 | 1.8 | 15.7 |
| **19-31 mo** | * | 7.2 | 8.3 | 10.6 | 11.8 | 12.9 | 14.0 | 1.8 | 17.1 |
| **20-32 mo** | * | 6.8 | 7.9 | 10.1 | 11.3 | 12.4 | 13.4 | 1.7 | 16.3 |
| **21-33 mo** | * | 6.8 | 7.9 | 10.1 | 11.3 | 12.4 | 13.4 | 1.7 | 16.3 |
| **22-34 mo** | * | 6.4 | 7.4 | 9.6 | 10.7 | 11.8 | 12.8 | 1.7 | 17.6 |
| **23-35 mo** | * | 6.1 | 7.1 | 9.2 | 10.3 | 11.2 | 12.2 | 1.5 | 16.6 |
| **24-36 mo** | * | 6.1 | 7.1 | 9.2 | 10.3 | 11.2 | 12.2 | 1.5 | 16.6 |
| **25-37 mo** | * | 6.1 | 7.1 | 9.2 | 10.3 | 11.2 | 12.2 | 1.5 | 16.6 |
| **26-38 mo** | * | 6.1 | 7.1 | 9.2 | 10.3 | 11.2 | 12.2 | 1.5 | 16.6 |
| **27-39 mo** | * | 5.8 | 6.7 | 8.7 | 9.7 | 10.7 | 11.6 | 1.5 | 17.6 |
| **28-40 mo** | * | 5.7 | 6.5 | 8.4 | 9.4 | 10.3 | 11.2 | 1.4 | 16.3 |
| **29-41 mo** | * | 5.7 | 6.5 | 8.4 | 9.4 | 10.3 | 11.2 | 1.4 | 16.3 |
| **30-42 mo** | * | 5.7 | 6.5 | 8.4 | 9.4 | 10.3 | 11.2 | 1.4 | 16.3 |
| **31-43 mo** | * | 5.7 | 6.5 | 8.4 | 9.4 | 10.3 | 11.2 | 1.4 | 16.3 |
| **32-44 mo** | * | 5.7 | 6.5 | 8.4 | 9.4 | 10.3 | 11.2 | 1.4 | 16.3 |
| **33-45 mo** | * | 5.5 | 6.3 | 8.1 | 9.1 | 9.9 | 10.7 | 1.4 | 17.1 |
| **34-46 mo** | * | 5.4 | 6.2 | 7.9 | 8.8 | 9.6 | 10.4 | 1.3 | 15.9 |
| **35-47 mo** | * | 5.4 | 6.2 | 7.9 | 8.8 | 9.6 | 10.4 | 1.3 | 15.9 |
| **36-48 mo** | * | 5.4 | 6.2 | 7.9 | 8.8 | 9.6 | 10.4 | 1.3 | 15.9 |
| **37-49 mo** | * | 5.4 | 6.2 | 7.9 | 8.8 | 9.6 | 10.4 | 1.3 | 15.9 |

**Supplemental Table 4 contd.**: Derived 12-month increments (unmodeled) estimates for length/height of the WHO- Tanner linear growth velocity reference.

| **Age Interval** | **-3 SND**^†^ | **-2 SND**^†^ | **-1 SND**^†^ | **Median** | **3 SND**^†^ | **2 SND**^†^ | **1 SND**^†^ | **SND**^†^ **(Median)** | **CV**^‡^ |
| --- | --- | --- | --- | --- | --- | --- | --- | --- | --- |
| **38-50 mo** | * | 5.4 | 6.2 | 7.9 | 8.8 | 9.6 | 10.4 | 1.3 | 15.9 |
| **39-51 mo** | * | 5.3 | 6.0 | 7.7 | 8.5 | 9.3 | 10.1 | 1.3 | 16.7 |
| **40-52 mo** | * | 5.1 | 5.9 | 7.5 | 8.3 | 9.0 | 9.8 | 1.2 | 15.6 |
| **41-53 mo** | * | 5.1 | 5.9 | 7.5 | 8.3 | 9.0 | 9.8 | 1.2 | 15.6 |
| **42-54 mo** | * | 5.1 | 5.9 | 7.5 | 8.3 | 9.0 | 9.8 | 1.2 | 15.6 |
| **43-55 mo** | * | 5.1 | 5.9 | 7.5 | 8.3 | 9.0 | 9.8 | 1.2 | 15.6 |
| **44-56 mo** | * | 5.1 | 5.9 | 7.5 | 8.3 | 9.0 | 9.8 | 1.2 | 15.6 |
| **45-57 mo** | * | 5.0 | 5.7 | 7.2 | 8.0 | 8.8 | 9.5 | 1.2 | 16.5 |
| **46-58 mo** | * | 4.9 | 5.6 | 7.0 | 7.8 | 8.5 | 9.2 | 1.1 | 15.5 |
| **47-59 mo** | * | 4.9 | 5.6 | 7.0 | 7.8 | 8.5 | 9.2 | 1.1 | 15.5 |
| **48-60 mo** | * | 4.9 | 5.6 | 7.0 | 7.8 | 8.5 | 9.2 | 1.1 | 15.5 |
| **49-61 mo** | * | 4.9 | 5.6 | 7.0 | 7.8 | 8.5 | 9.2 | 1.1 | 15.5 |
| **50-62 mo** | * | 4.9 | 5.6 | 7.0 | 7.8 | 8.5 | 9.2 | 1.1 | 15.5 |
| **51-63 mo** | * | 4.7 | 5.4 | 6.8 | 7.6 | 8.3 | 9.0 | 1.1 | 16.4 |
| **52-64 mo** | * | 4.7 | 5.3 | 6.7 | 7.5 | 8.1 | 8.8 | 1.0 | 15.2 |
| **53-65 mo** | * | 4.7 | 5.3 | 6.7 | 7.5 | 8.1 | 8.8 | 1.0 | 15.2 |
| **54-66 mo** | * | 4.7 | 5.3 | 6.7 | 7.5 | 8.1 | 8.8 | 1.0 | 15.2 |
| **55-67 mo** | * | 4.7 | 5.3 | 6.7 | 7.5 | 8.1 | 8.8 | 1.0 | 15.2 |
| **56-68 mo** | * | 4.7 | 5.3 | 6.7 | 7.5 | 8.1 | 8.8 | 1.0 | 15.2 |
| **57-69 mo** | * | 4.6 | 5.3 | 6.6 | 7.3 | 8.0 | 8.6 | 1.1 | 16.1 |
| **58-70 mo** | * | 4.5 | 5.2 | 6.5 | 7.2 | 7.8 | 8.4 | 1.0 | 15.0 |
| **59-71 mo** | * | 4.5 | 5.2 | 6.5 | 7.2 | 7.8 | 8.4 | 1.0 | 15.0 |
| **60-72 mo** | * | 4.5 | 5.2 | 6.5 | 7.2 | 7.8 | 8.4 | 1.0 | 15.0 |

* Values not available to derive
^†^ SND: standard normal deviate also defined as 1 z-score

^‡^ CV: Coefficient of variation

1. Supplemental Figure 1: WHO-Tanner sex-combined derived referent median scatterplot

and modeled 12-month linear velocities from birth through 59 months of age ^*†^


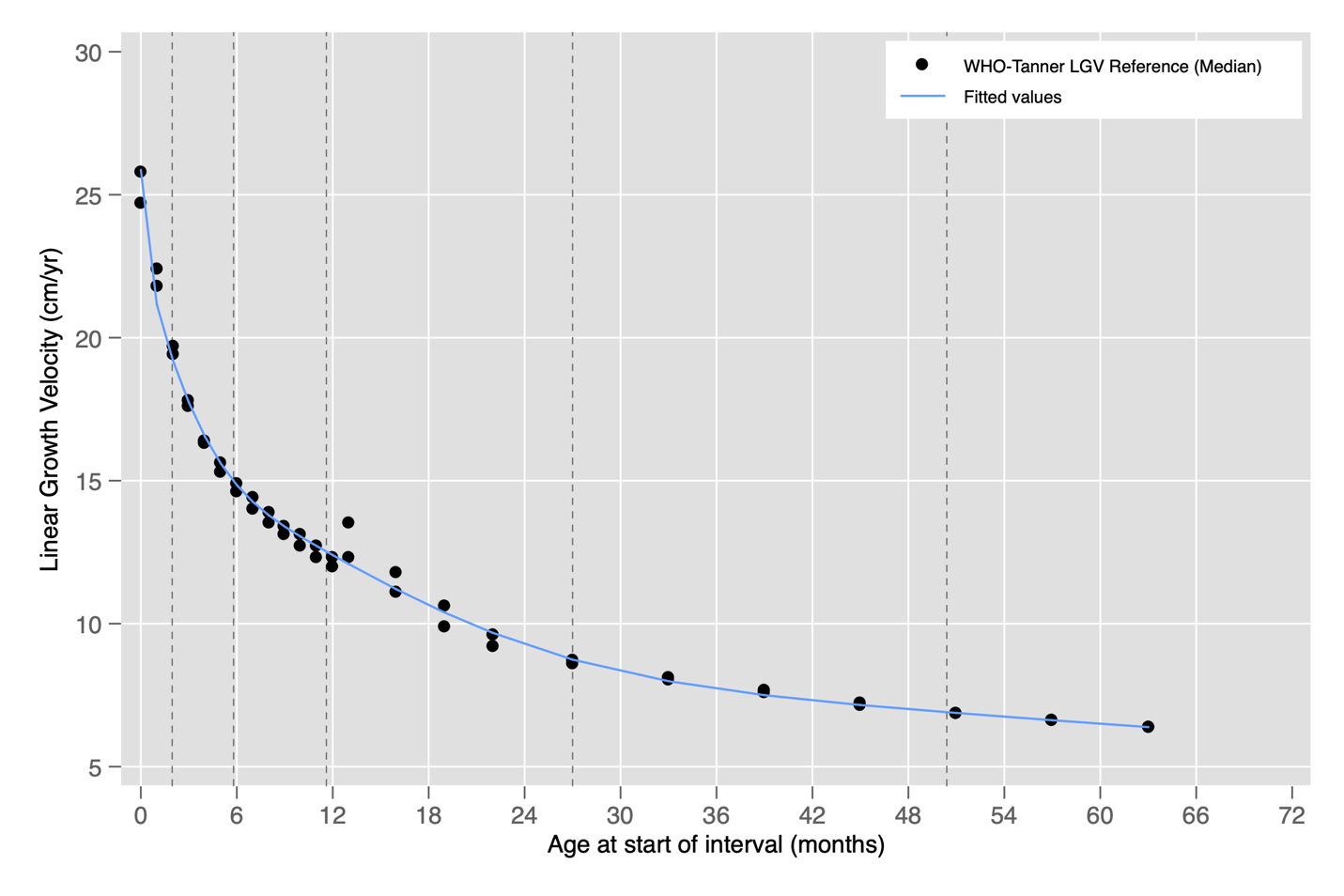


^*^ Age at the start of the interval
^†^ Generated using a cubic-restricted spline model with knots (dotted horizontal lines) at 2, 6, 12, 27 and 50 months of age:

E[Y_p50_] = $B$_0_ + $B$_i1age_ (X -$\tau$_i_) + $B$_i2age_ (X -$\tau$_i_)^2^ + $B$_i3age_ (X -$\tau$_i_)^3^ + $B$_i4age_ (X -$\tau$_i_)^4^ + $B$_5_x_i5sex_ + $B$_6_x_i5sex_x_i1age_ + $B$_7_x_i5sex_x_i2age_ + $B$_8_x_i5sex_x_i3age_ + $B$_7_x_i9sex_x_i4age_ + $\varepsilon$

1. Supplemental Figure 2: WHO-Tanner modeled sex-combined linear growth velocity reference curve through 59 months of age ^* †^ (median + 2 standard normal deviate or z-score).


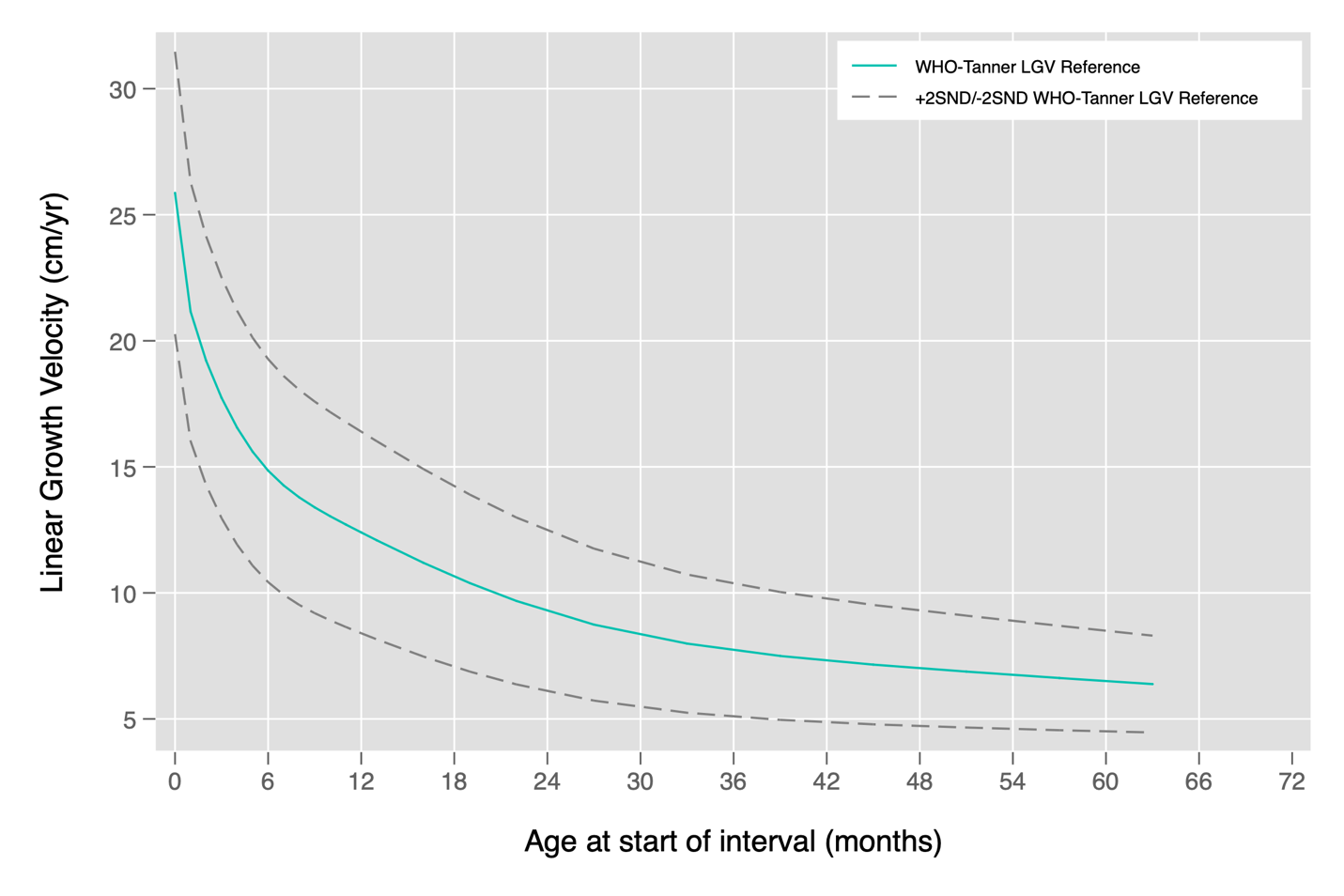


^*^ Age at the start of interval ^†^ Generated using a cubic-restricted spline model with knots at 2, 6, 12, 27 and 50 months of age:

E[Y_p50_] = $B$_0_ + $B$_i1age_ (X -$\tau$_i_) + $B$_i2age_ (X -$\tau$_i_)^2^ + $B$_i3age_ (X -$\tau$_i_)^3^ + $B$_i4age_ (X -$\tau$_i_)^4^ + $B$_5_x_i5sex_ + $B$_6_x_i5sex_x_i1age_ + $B$_7_x_i5sex_x_i2age_ + $B$_8_x_i5sex_x_i3age_ + $B$_7_x_i9sex_x_i4age_ + $\varepsilon$

1. Supplemental Figure 3: WHO-Tanner sex-specific derived variance in linear growth velocity scatterplot and modeled variance in linear growth velocity through 59 months of age *^†^


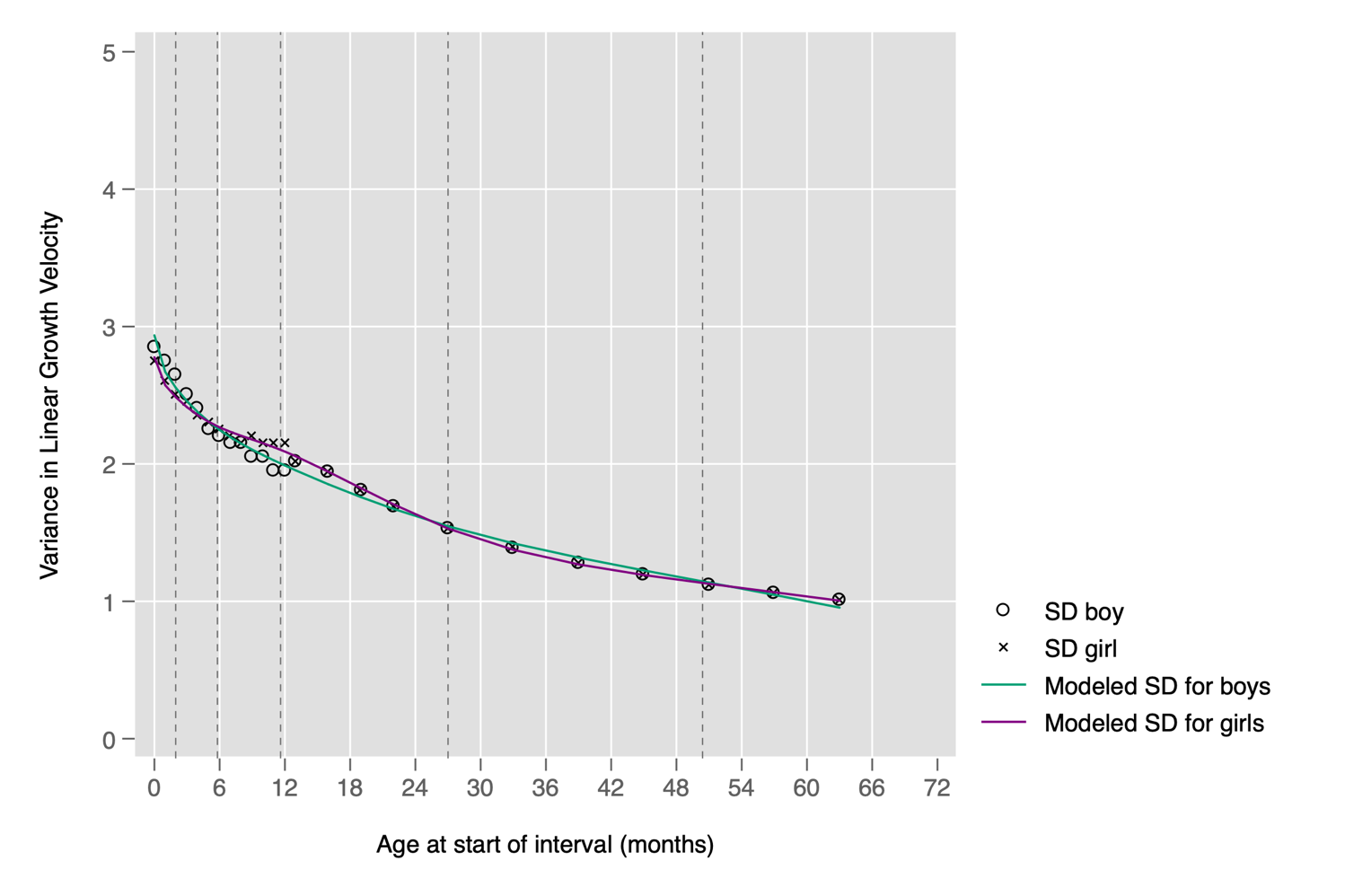


^*^ Age at the start of the interval
^†^ Generated using a cubic-restricted spline model with knots (horizontal dotted lines) at 2, 6, 12, 27 and 50 months of age:

E[Y_p50_] = $B$_0_ + $B$_i1age_ (X -$\tau$_i_) + $B$_i2age_ (X -$\tau$_i_)^2^ + $B$_i3age_ (X -$\tau$_i_)^3^ + $B$_i4age_ (X -$\tau$_i_)^4^ + $B$_5_x_i5sex_ + $B$_6_x_i5sex_x_i1age_ + $B$_7_x_i5sex_x_i2age_ +

$B$_8_x_i5sex_x_i3age_ + $B$_7_x_i9sex_x_i4age_ + $\varepsilon$

1. Supplemental Figure 4: Study Flow diagram for inclusion into the analytic sample for 0-59 month old Nepali children from the Tarai from whom measurements were taken between 2013- 2016

1. Baumgartner RN, Roche AF & Himes JH (1986) Incremental growth tables: Supplementary to previously published charts. Am. J. Clin. Nutr. 43, 711–722. [↑](#footnote-ref-1)
2. Roche AF, Guo S & Moore WM (1989) Weight and recumbent length from 1 to 12 mo of age: Reference data for 1-mo increments. *Am. J. Clin. Nutr.* **49**, 599–

   607. [↑](#footnote-ref-2)
3. Guo S, Roche AF, Fomon SJ, et al. (1991) Reference data on gains in weight and length during the first two years of life. J. Pediatr. 119, 355–362. [↑](#footnote-ref-3)
4. Tanner JM, Whitehouse RH & Takaishi M (1966) Standards from birth to maturity for height, weight, height velocity, and weight velocity: British children, 1965 Part II. Arch. Dis. Child. 41, 613–635 [↑](#footnote-ref-4)
5. Prader A, Largo RH, Molinari L, et al. (1989) Physical growth of Swiss children from birth to 20 years of age. First Zurich longitudinal study of growth and development. Helv Paediatr Acta Suppl. 52, 1–125 [↑](#footnote-ref-5)
6. WHO Multicentre Growth Reference Study Group (2009) WHO Child Growth Standards: Growth velocity based on weight, length and head circumference. Methods and development. Geneva. [↑](#footnote-ref-6)
7. Baumgartner RN, Roche AF & Himes JH (1986) Incremental growth tables: Supplementary to previously published charts. Am. J. Clin. Nutr. 43, 711–722. [↑](#footnote-ref-7)
8. Guo S, Roche AF, Fomon SJ, et al. (1991) Reference data on gains in weight and length during the first two years of life. J. Pediatr. 119, 355–362. [↑](#footnote-ref-8)
9. Tanner JM, Whitehouse RH & Takaishi M (1966) Standards from birth to maturity for height, weight, height velocity, and weight velocity: British children, 1965 Part II. Arch. Dis. Child. 41, 613–635 [↑](#footnote-ref-9)
10. Prader A, Largo RH, Molinari L, et al. (1989) Physical growth of Swiss children from birth to 20 years of age. First Zurich longitudinal study of growth and development. Helv Paediatr Acta Suppl. 52, 1–125 [↑](#footnote-ref-10)
11. WHO Multicentre Growth Reference Study Group (2009) WHO Child Growth Standards: Growth velocity based on weight, length and head circumference. Methods and development. Geneva. [↑](#footnote-ref-11)
