# Supplementary figures and images for: Evaluating preschool linear growth velocities: an interim reference illustrated in Nepal
Source: Public Health Nutr. 2023 Nov 7;26(12):2704–16. doi: 10.1017/S1368980023002409 (PMC10755438; doi:10.1017/S1368980023002409)

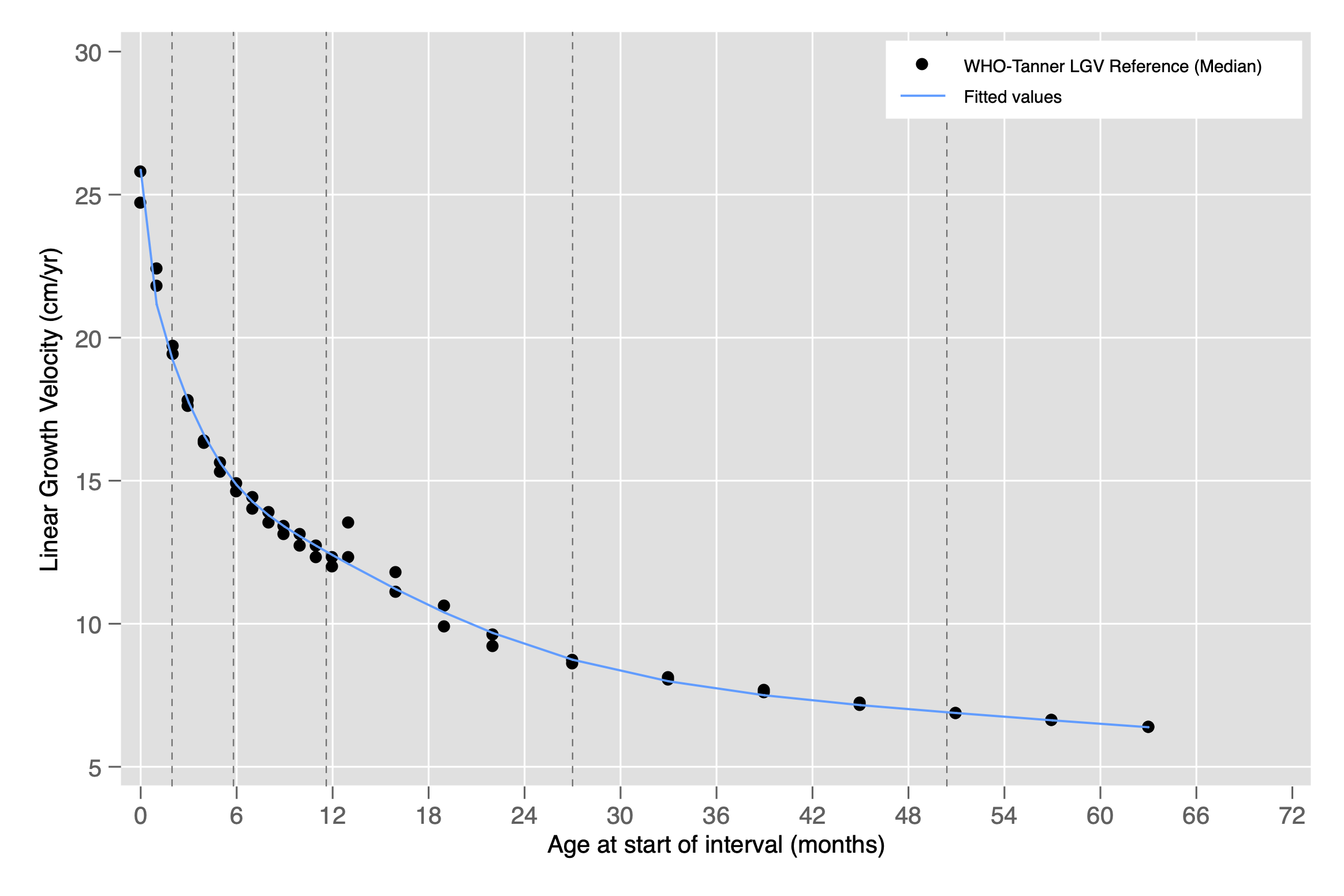

Supplement: Manohar et al. supplementary material 2 — Manohar et al. supplementary material [file S1368980023002409sup002.zip › SuppFig 1.tif]

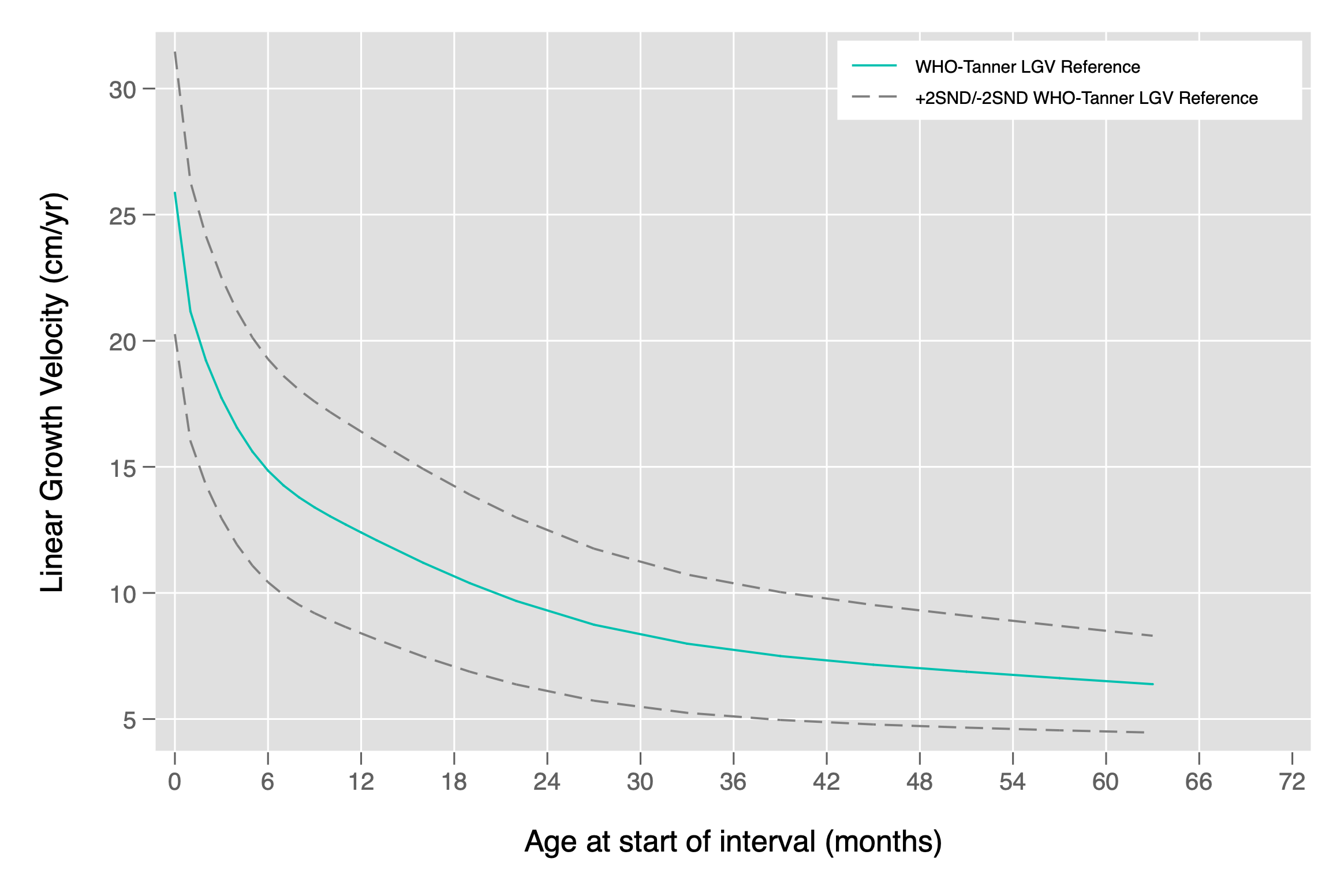

Supplement: Manohar et al. supplementary material 3 — Manohar et al. supplementary material [file S1368980023002409sup003.zip › SuppFig 2.tif]

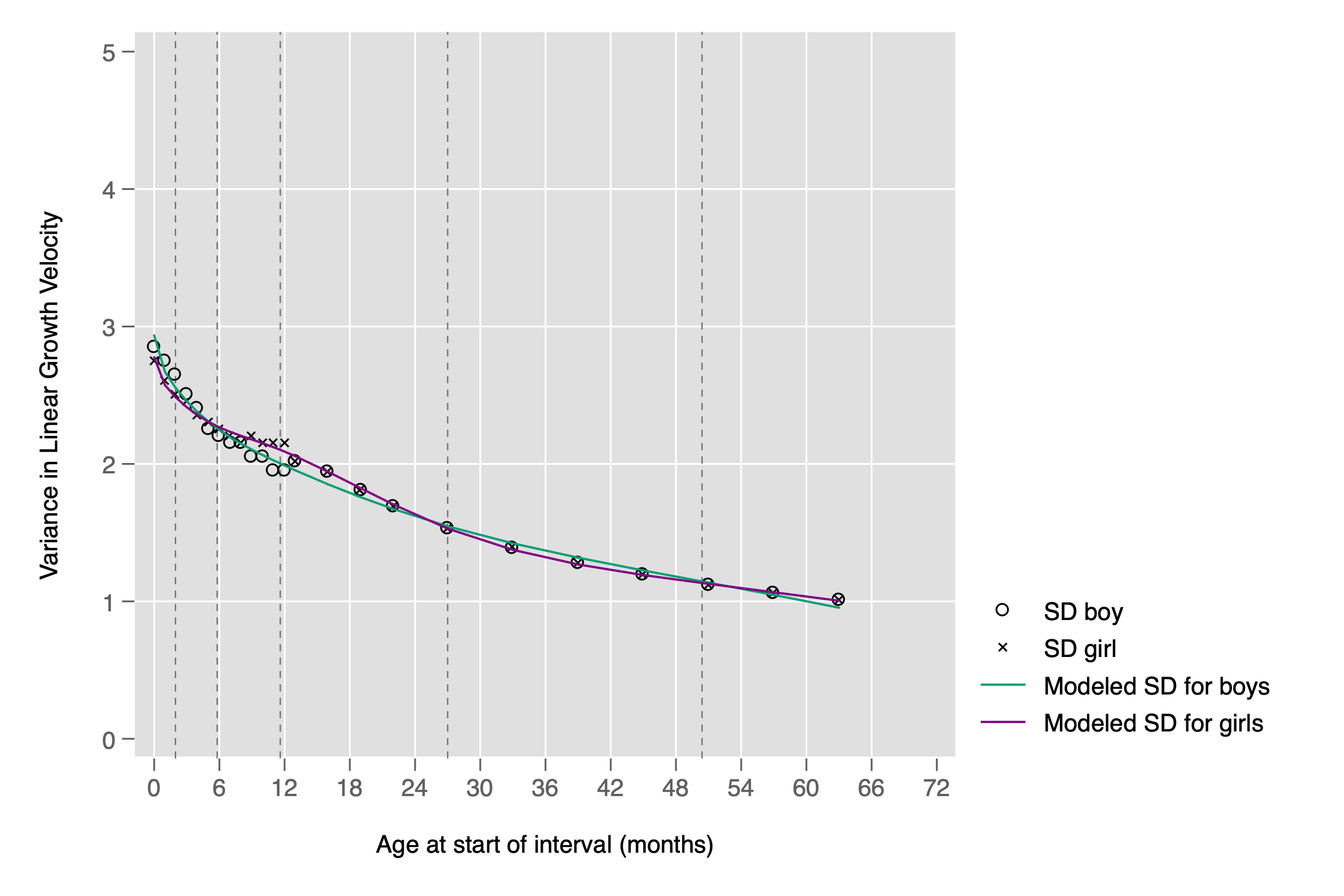

Supplement: Manohar et al. supplementary material 4 — Manohar et al. supplementary material [file S1368980023002409sup004.zip › SuppFig 3.tif]

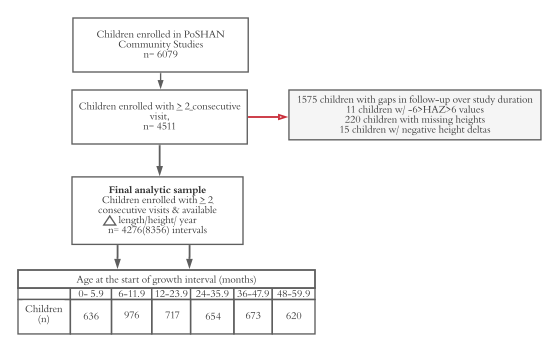

Supplement: Manohar et al. supplementary material 5 — Manohar et al. supplementary material [file S1368980023002409sup005.zip › SuppFig 4.png]
